# Sericin coated thin polymeric films reduce keratinocyte proliferation via the mTOR pathway and epidermal inflammation through IL17 signaling in psoriasis rat model

**DOI:** 10.1038/s41598-023-39218-y

**Published:** 2023-07-26

**Authors:** Pornanong Aramwit, Kamonpan Fongsodsri, Khwanchanok Tuentam, Onrapak Reamtong, Tipparat Thiangtrongjit, Tapanee Kanjanapruthipong, Vamsi K. Yadavalli, Sumate Ampawong

**Affiliations:** 1 Bioactive Resources for Innovative Clinical Applications Research Unit, Department of Pharmacy Practice, Faculty of Pharmaceutical Sciences, Chulalongkorn University, Phayathai Road, Pathumwan, Bangkok, 10330 Thailand; 2 The Academy of Science, The Royal Society of Thailand, Dusit, Bangkok, 10330 Thailand; 3 Department of Tropical Pathology, Faculty of Tropical Medicine, Mahidol University, 420/6 Ratchawithi Road, Ratchathewi, Bangkok, 10400 Thailand; 4 Department of Molecular Tropical Medicine and Genetics, Faculty of Tropical Medicine, Mahidol University, Ratchawithi Road, Ratchathewi, Bangkok, 10400 Thailand; 5 Department of Chemical and Life Science Engineering, Virginia Commonwealth University, 601 W Main Street, Richmond, VA 23284 USA

**Keywords:** Experimental models of disease, Pharmacology

## Abstract

Therapeutic treatment forms can play significant roles in resolving psoriatic plaques or promoting wound repair in psoriatic skin. Considering the biocompatibility, mechanical strength, flexibility, and adhesive properties of silk fibroin sheets/films, it is useful to combine them with anti-psoriatic agents and healing stimulants, notably silk sericin. Here, we evaluate the curative properties of sericin-coated thin polymeric films (ScF) fabricated from silk fibroin, using an imiquimod-induced psoriasis rat model. The film biocompatibility and psoriatic wound improvement capacity was assessed. A proteomics study was performed to understand the disease resolving mechanisms. Skin-implantation study exhibited the non-irritation property of ScF films, which alleviate eczema histopathology. Immunohistochemical and gene expression revealed the depletion of β-defensin, caspase-3 and -9, TNF-α, CCL-20, IL-1β, IL-17, TGF-β, and Wnt expressions and *S100a14* mRNA level. The proteomics study suggested that ScF diminish keratinocyte proliferation via the mTOR pathway by downregulating mTOR protein, corresponding to the modulation of TNF-α, Wnt, and IL-1β levels, leading to the enhancement of anti-inflammatory environment by IL-17 downregulation. Hematology data demonstrated the safety of using these biomaterials, which provide a potential therapeutic-option for psoriasis treatment due to desirable effects, especially anti-proliferation and anti-inflammation, functioning via the mTOR pathway and control of IL-17 signaling.

## Introduction

Psoriasis is a chronic, non-contagious, and T cell-mediated autoinflammatory skin disease, which manifests in the form of macroscopic red and scaly plaque and microscopic epidermal lesions, particularly hyperkeratosis, acanthosis, dermatitis, and folliculitis. A single treatment cannot provide the best effective therapeutic outcome. In addition, most treatments cannot be used for prolonged periods of time, and need to be used in rotation to minimize adverse effects. Research to devise new therapeutic regimens has been ongoing for several decades, and the current treatment armamentarium includes topical preparations, ultraviolet therapy, and systemic therapies. The choice of suitable treatment methods depends on disease pattern and severity, comorbidities, patient preference, and other related factors such as the presence of complicated wounds in patients with psoriatic skin conditions. This includes for example, individuals who have undergone trauma, infection, or surgery.

Different types of psoriatic lesions, with or without associated complications, require appropriate treatment and care. Many adjunctive therapeutic dressings, each associated with their own advantages and disadvantages, have been developed to enhance treatment outcomes. Occlusion therapy with colloidal dressings can be used for some patterns of psoriasis, such as palmoplantar, localized pustular, and pustulosis psoriasis, to enhance the epidermal absorption of active ingredients^1^. The combination treatment of hydrocolloid dressing, a hydrated-gel like matrix, with anti-psoriatic cream-based preparations such as corticosteroids or calcitriol presents some benefits such as ease of application, patient acceptability, relief from itching and traumatic plaque, limited use of steroids, and cost effectiveness^1–3^. However, bacterial contamination with long-term use of the occlusive materials is an important contraindication^1^. Presently, dual purpose dressings for simultaneous alleviation of psoriatic skin conditions and facilitating wound healing are being developed.

Sericin and fibroin, silk proteins derived from *Bombyx mori* cocoons, are natural biopolymers with several medicinal properties, and are especially known to enhance wound healing^4^. Recently, we have demonstrated the efficacy of cream-based as well as polyvinyl alcohol-based preparations of sericin for treating psoriasis in preclinical studies^5,6^. The main mechanisms of anti-psoriatic action of sericin occur via the inhibition of epidermal cell overgrowth, epidermal inflammation, and imbalance of epidermal cell homeostasis, through immunomodulative and antioxidative effects.

Two-dimensional (2D) silk protein (specifically silk fibroin) polymeric films and membranes have been explored for various biomedical applications including drug delivery, biofiltration, biosensors, biomimetic cellular construction, wearable devices, smart skin, and tissue regeneration^7–10^. Silk films are flexible and mechanically robust, and can also be formed in micropatterned configurations via photolithography. Owing to desirable properties such as mechanical flexibility, adhesion, biocompatibility, and non-toxic nature^7^, 2D fibroin sheets can potentially be combined with bioactive agents such as sericin, for treating psoriasis. Being biodegradable under proteolytic conditions, these films can also form a sustainable, zero-waste system.

Therefore, based on an in vivo approach—“imiquimod-induced psoriasis rat model,” this study aims to examine the therapeutic properties, toxic effects, and disease-resolving mechanisms of sericin-coated thin fibroin films for psoriasis treatment. Histopathology, immunohistochemistry, RT-qPCR (quantitative reverse transcription polymerase chain reaction), and proteomics were performed in the study. This study provides an alternative therapeutic form ScF—a combination of sericin extraction and flexible, photocrosslinked 2D silk fibroin films, for application in psoriasis treatment. Study outcomes may be useful for further development of a new prototype treatment which can be evaluated in clinical trials.

## Results

### Composition of amino acids in sericin extraction and non-irritability of sericin coated films (ScF)

**Figure 1:**
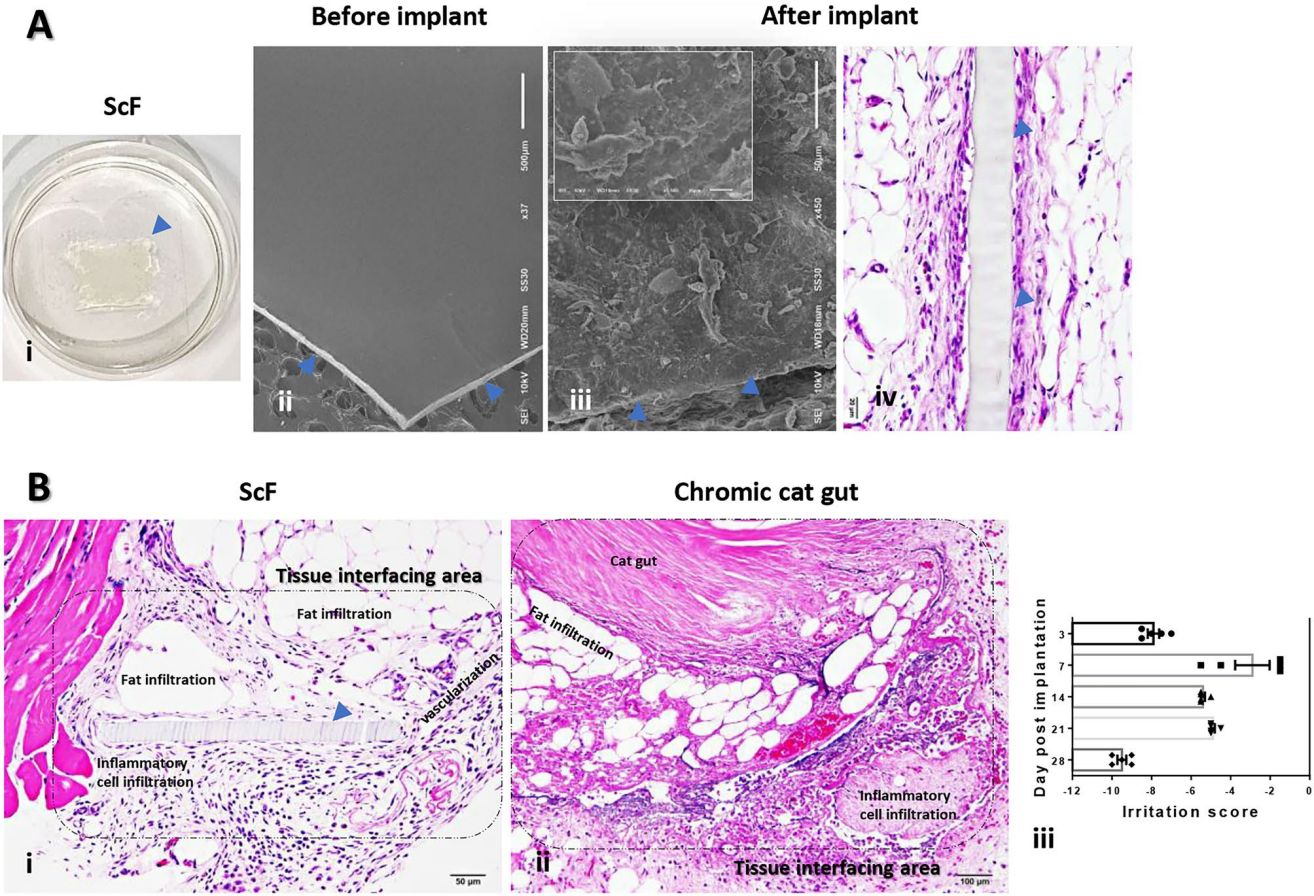
Bioimplantation study: (**A**) 5% sericin-coated thin polymeric film fabricated from fibroin (ScF) (i), electron micrographs and histological image before (ii) and after (iii–iv) implantations for 3 days (arrow-head; material), (**B**) Histomorphological appearance of tissue interfacing area in ScF (i) and Chromic cat gut (ii) after implantation for 3 days with represented in a bar graph along with the irritation score (iii).

The amino acid composition of sericin was evaluated to be: Serine (29.66%), aspartic acid (18.06%), glycine (10.01%), threonine (8.56%), glutamic acid (6.65%), tyrosine (6.35%), arginine (6.35%), lysine (4.02%), valine (3.94%), alanine (3.81%), and leucine (2.54%). The sericin was coated on photocrosslinked fibroin films to form sericin-coated films (ScF) (Fig. 1Ai). The use of photocrosslinking allows the formation of mechanically robust, flexible thin films of controllable thickness, and potentially, micropatterned films for targeted surface treatments^7^.

A bioimplantation study was performed to demonstrate the positive response of host immune system when it comes in contact with ScF, as compared to Chromic catgut. Electron micrographs demonstrated an ultrastructure of ScF, interacting with host cell infiltration predominately, fibroblasts and inflammatory cells during implantation (Fig. 1Aii–iv). Histopathological evaluation focused on the extent of host inflammatory responses and tissue alterations in the tissue interfacing area (Fig. 1Bi–ii). The immunity reaction of the host was lesser with ScF as compared to the control. Therefore, the final irritation score demonstrated the non-irritation property of ScF during all periods of implantation (Fig. 1Biii).

### ScF reduces the main histopathological lesions in psoriasis

**Figure 2:**
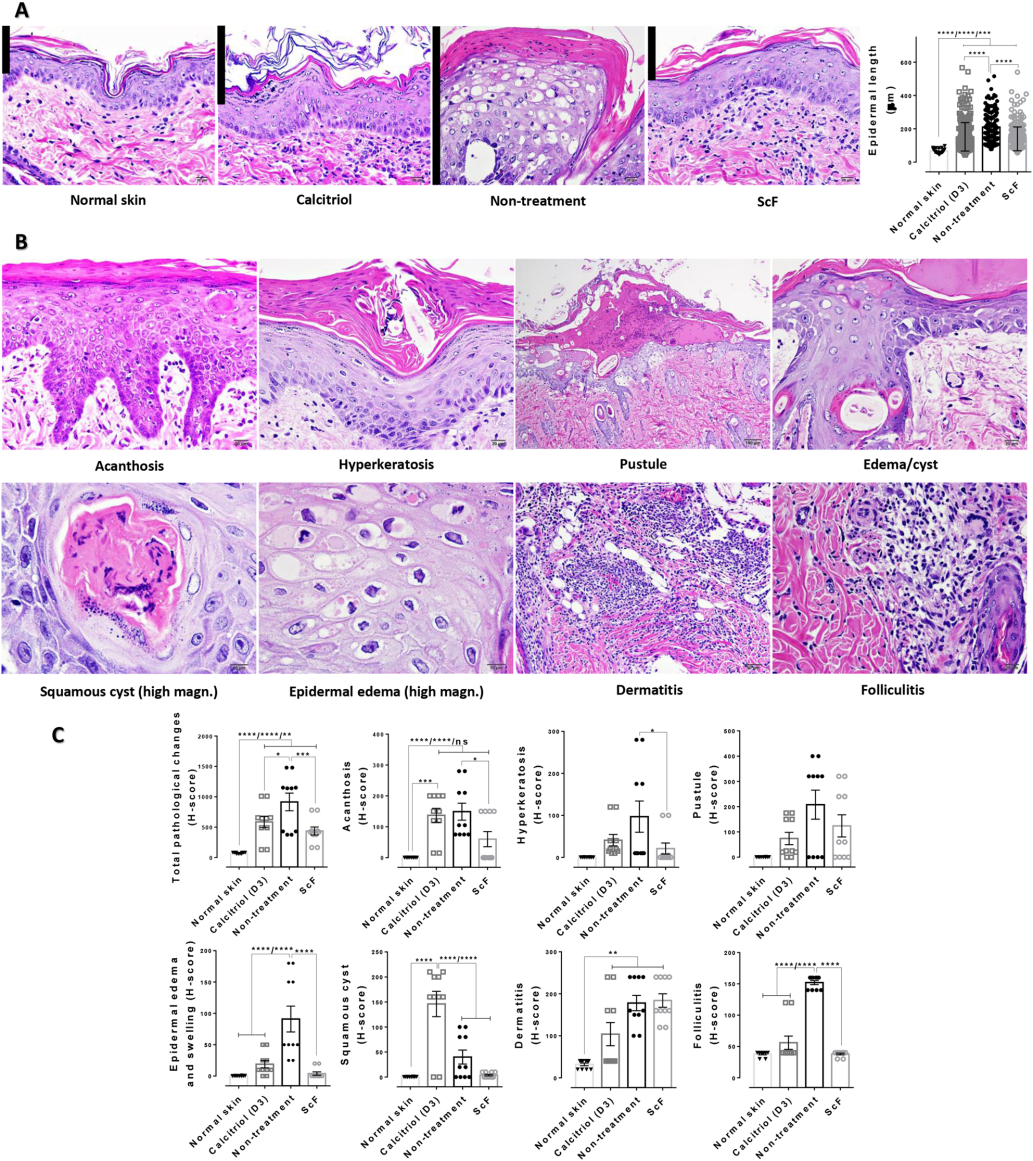
Histopathological lesions and severity score in psoriasis rats with any treatment: (**A**) Histopathological images of epidermal thickening and epidermal length in any treatment, compared with normal skin, (**B**) Main histopathological changes in imiquimod-induced psoriasis; an elongation of rete ridges extended to dermis (acanthosis), a thickening of the stratum corneum (hyperkeratosis), a deposition of exudate including tissue debris and inflammatory cells, particularly neutrophil (pustule), an increase in the size of epidermal cell (edema), a formation of cysts in the dermis, with a perforating epidermal component (squamous cyst), an infiltration of inflammatory cells in the dermis (dermatitis) or the hair follicle (folliculitis), (**C**) A bar graph of epidermal histopathological score in various skin conditions such as acanthosis, hyperkeratosis, pustule, epidermal edema and swelling, squamous cyst, dermatitis, and folliculitis in comparison with normal skin.

Epidermal cell overgrowth is one of the main histopathological changes in imiquimod-induced psoriasis rat models. Compared with the non-treatment group, psoriatic rats which received ScF and calcitriol had significantly reduced epidermal length (Fig. 2A). Other epidermal histopathological lesions were also examined (Fig. 2B). The overall score was reduced in rats treated with ScF and calcitriol, compared with rats which were not treated with ScF and calcitriol. Similar to ScF, calcitriol-treated group also exhibited a lower score of epidermal edema and folliculitis than the non-treatment group. The proportion of acanthosis, hyperkeratosis, and squamous cysts was lowered in ScF-treated group, as compared to calcitriol and non-treated groups. However, pustule formation tended to be declined in ScF- and calcitriol-treated rats. In addition, dermatitis was still observed in rats administered with both ScF and calcitriol.

### Anti-psoriatic properties of ScF based on immunohistochemical study

**Figure 3:**
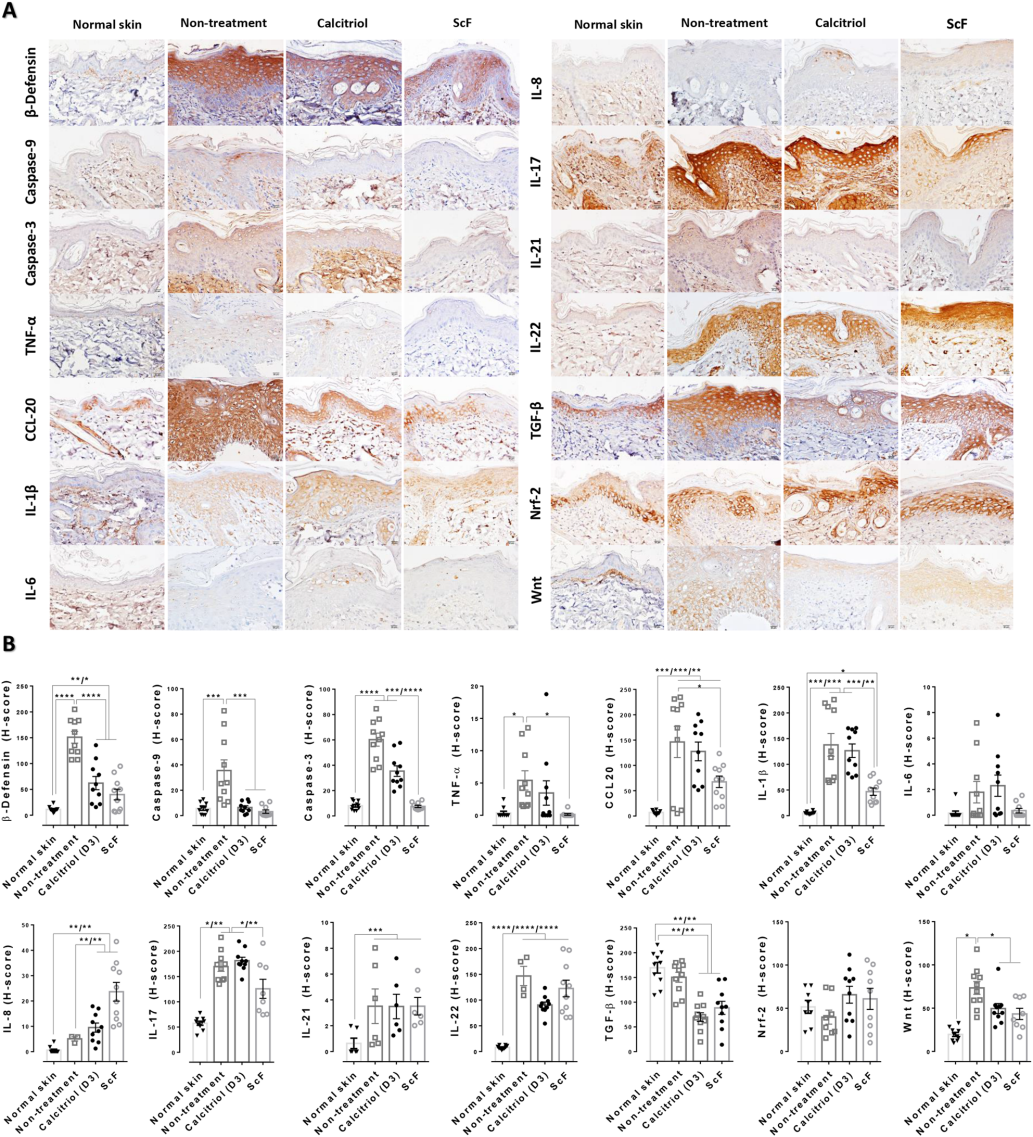
Immunohistochemical staining and score of psoriasis-related markers in any treatment: (**A**) Epidermal immunolocalization of β-defensin, caspase-9 and -3, TNF-α, CCL20, IL-1β, IL-6, IL-8, IL-17, IL-21, IL-22, TGF-β, Nrf-2, and Wnt in any treatment, (**B**) A bar graph comparison of the immunolabeled score (H-score) of psoriasis-related markers in any treatment.

The expressions of psoriatic-related protein (an antimicrobial peptide), apoptotic marker, proinflammatory cytokine, chemokines, anti-inflammatory cytokine, antioxidation protein, and antiproliferation marker were examined using immunohistochemical staining (Fig. 3). Immunolabeling of β-defensin, caspase-3 and -9, and Wnt was observed to be reduced in rats treated with ScF and calcitriol compared with non-treated rats. The ScF treatment group exhibited a reduction in TNF-α, CCL-20, IL-1β, and IL-17, when compared with calcitriol and non-treatment groups. However, the expression of IL-6 and Nrf-2 was not significantly different in all groups. TGF-β was lowered in both treatment groups, as compared to non-treated rats and normal skin. Nonetheless, IL-8 expression was increased in the ScF treatment group when compared to calcitriol- and non-treatment groups. The expressions of β-defensin, caspase-3 and -9, Wnt, TNF-α, CCL-20, IL-1β, IL-8, IL-17, IL-21, and IL-22 in normal skin were lower than present in other groups.

### Hematology and blood clinical chemistry

For the toxicological assessment of ScF application 7 days post-treatment, hematological and blood clinical chemistry was examined (Table 1). All groups demonstrated a marked increase in WBC (white blood cells), RDW (red cell distribution width), RET (reticulocyte count test), eosinophils, basophils, and SGPT (serum glutamic pyruvic transferase). A slight increase in MCHC (mean corpuscular hemoglobin concentration) and MPV (mean platelet volume) was observed. They also exhibited a mild reduction in RBC (red blood cells), HGB (hemoglobin), HCT (hematocrit), MCV (mean corpuscle volume), and PCT (plateletcrit). However, monocyte depletion was markedly observed. It is worth noting that non-treated rats had a markedly high number of neutrophils, while calcitriol-treated rats had increased SGPT and SGOT (serum glutamic-oxaloacetic transaminase) levels when compared with other groups.

**Table 1:** Hematological and blood clinical chemistry in rat-treated with or without ScF.

Parameter	Normal limits^#^	Complete blood count and liver and kidney profiles (Mean ± SD)		
		ScF	Calcitriol	Non-treatment
WBC (10^6^/µl)	4.23 ± 0.72	8.63 ± 6.76^↑^	8.25 ± 1.51^↑^	11.81 ± 5.21^↑^
RBC (10^6^/µl)	9.27 ± 0.63	6.00 ± 2.35^↓^	7.52 ± 0.50^↓^	7.43 ± 0.61^↓^
HGB (g/dl)	17.78 ± 1.02	11.57 ± 3.82^↓^	14.18 ± 0.86^↓^	13.82 ± 0.83^↓^
HCT (%)	56.45 ± 3.80	33.5 ± 14.20^↓^	42.30 ± 2.70^↓^	41.30 ± 2.83^↓^
MCV (fl)	60.93 ± 1.75	54.65 ± 3.93^↓^	56.26 ± 2.26^↓^	55.62 ± 2.76^↓^
MCH (pg)	19.20 ± 0.51	20.00 ± 2.40	18.86 ± 0.58	18.60 ± 0.88
MCHC (g/dl)	31.50 ± 0.38	36.97 ± 7.62^↑^	33.50 ± 0.51^↑^	33.48 ± 0.54^↑^
PLT (10^3^/µl)	804.50 ± 136.88	559.50 ± 343.19	728.60 ± 123.32	624.40 ± 123.32
RDW (%)	17.79 ± 1.94	28.35 ± 2.56^↑^	27.82 ± 1.53^↑^	28.08 ± 1.11^↑^
PDW (fl)	8.41 ± 0.45	8.25 ± 0.34	8.88 ± 0.33	8.90 ± 0.33
RET (K/µl)	232.88 ± 53.78	342.10 ± 204.17^↑^	450.92 ± 119.98^↑^	405.46 ± 49.06^↑^
MPV (fl)	7.63 ± 0.69	8.52 ± 0.59^↑^	8.74 ± 0.58^↑^	8.92 ± 0.32^↑^
PCT (%)	0.61 ± 0.08	0.48 ± 0.31^↓^	0.62 ± 0.14	0.55 ± 1.00
Neutrophils (%)	11.73 ± 6.98	6.95 ± 8.27	10.90 ± 11.06	22.14 ± 13.15*^↑^
Lymphocytes (%)	79.88 ± 7.03	82.72 ± 7.83	74.82 ± 15.05	66.68 ± 13.52
Eosinophils (%)	1.17 ± 0.33	8.35 ± 3.79^↑^	12.08 ± 7.16^↑^	9.42 ± 2.29^↑^
Basophils (%)	0.35 ± 0.38	1.95 ± 1.27^↑^	2.04 ± 1.35^↑^	1.68 ± 0.76^↑^
Monocytes (%)	6.87 ± 1.49	0.02 ± 0.05^↓^	0.16 ± 0.25^↓^	0.08 ± 0.08^↓^
Blood urea nitrogen (mg/dl)	20.73 ± 2.51	16.97 ± 6.76	15.84 ± 5.50	11.98 ± 9.31
Creatinine (mg/dl)	0.67 ± 0.04	0.17 ± 0.06	0.14 ± 0.08	0.08 ± 0.11
SGPT (U/l)	53.18 ± 10.15	130.67 ± 65.33^↑^	265.56 ± 329.42*^↑^	100.22 ± 32.82^↑^
SGOT (U/l)	93.73 ± 11.96	34.37 ± 13.40^↓^	49.58 ± 35.48*^↓^	21.54 ± 15.28^↓^

*WBC* white blood cell, *RBC* red blood cell, *HCT* hematocrit, *MCV* mean corpuscular volume, *MCH* mean corpuscular hemoglobin, *MCHC* mean corpuscular hemoglobin concentration, *PLT* platelet, *RDW* red cell distribution width, *SGPT* serum pyruvate-glutamate transaminase, *SGOT* serum glutamate–oxaloacetate transaminase.

*p value < 0.05 or **p value < 0.01; significant difference between any treatment group.

^#^In-house value from animal supplier.

^↑^ or ^↓^increase or decrease when comparing with normal ranges, respectively.

### Effect of ScF on genes involved psoriasis

**Figure 4:**
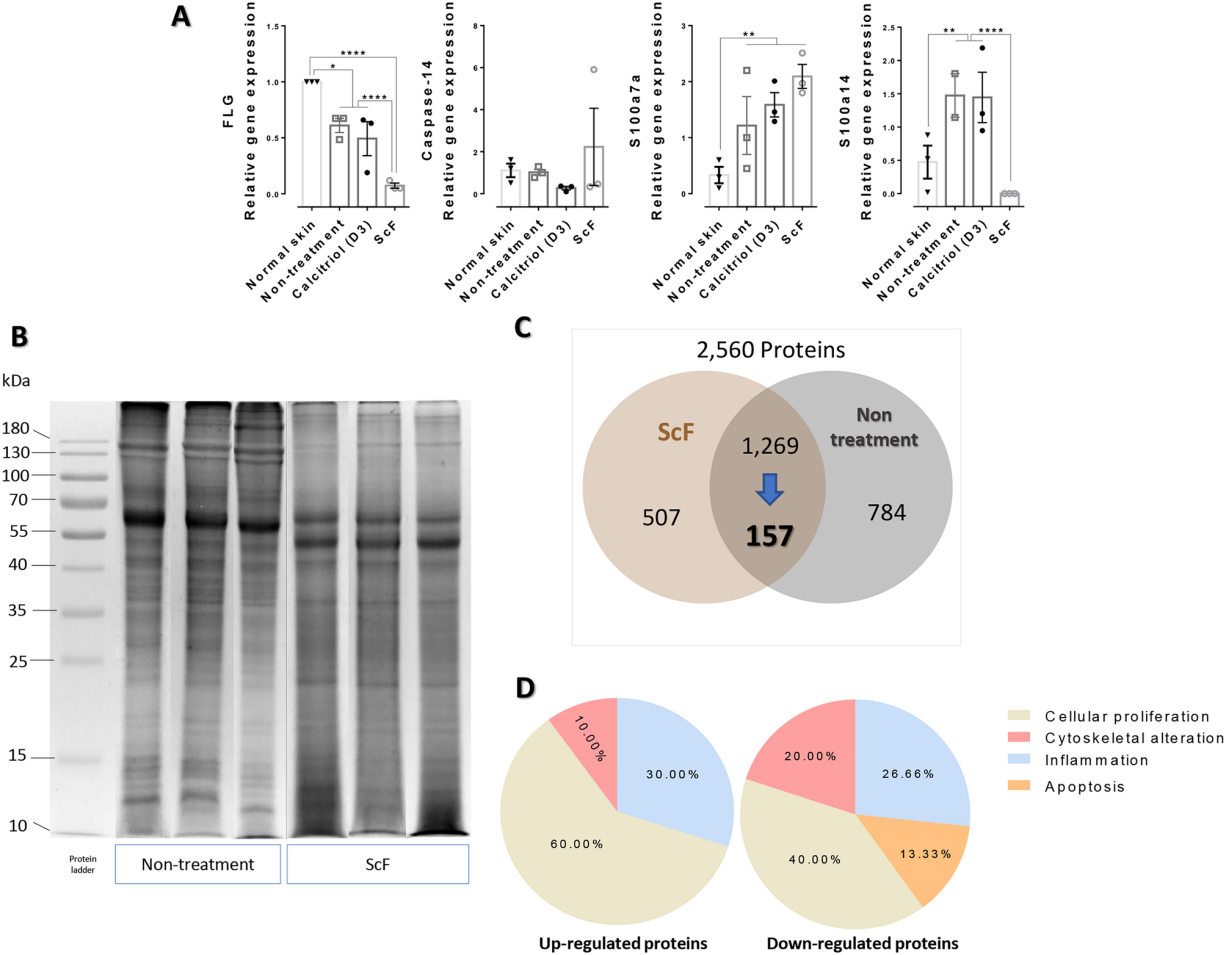
The expression of the gene involved with psoriasis and label-free quantitative proteomics analysis: (**A**) A bar graph comparison of mRNA expression of gene-related psoriasis markers (*FLG*, *caspase-14*, *S100a7a*, and *S100a14* in any treatment, (**B**) SDS-PAGE gel of psoriatic skin in rats treated with and without ScF. Each lane represents triplicate samples of ScF and non-treatment groups. Full-length gels are deposited in the Supplementary Fig. S2. (**C**) An illustration of protein differentiation in psoriatic skin-treated with or without ScF, (**D**) Protein characterization according to the 4 main pathogeneses of psoriasis (abnormal cellular proliferation, cytoskeletal alteration, auto-inflammation, and apoptosis) in 157 significant fold-changed proteins.

PT-qPCR data demonstrated that ScF-treated rats and normal skin presented a marked reduction in *S100a14* gene expression, compared to other groups (Fig. 4A). However, the gene expression level of *FLG* in ScF- and calcitriol-treated rats was lower than that in the non-treatment group and normal skin. *S100a7a* gene expression in normal skin were lower than other groups. In addition, the mRNA levels of *caspase-14* and *S100a7a* were not significantly different across all groups.

### Proteomics study

#### Protein identification in psoriatic rat skin with and without ScF treatment

To determine the alteration of epidermal proteins affected by ScF treatment in a psoriasis rat model, a proteomics study was performed. A total of 2560 proteins were identified—507 proteins were found only in ScF-treated rats, while 784 proteins were present in non-treated rats. 1269 proteins observed in both groups, and a significant alteration was observed in the expression of 157 proteins in ScF-treated and non-treated rats. All the 157 up- and down-regulated proteins are shown in Tables S3 and S4, respectively as Supplementary Files. In addition, at the fold-change cutting point at ≥ 2.0, 17 up- and 38 down-expressed proteins were identified from these 157 proteins (Fig. 4B,C).

#### Protein categorization along the main pathogenesis of psoriasis

There are 4 major pathways that play an important role in the pathogenesis of psoriasis: (1) inflammation, (2) epidermal cell apoptosis, (3) cellular hyper-proliferation, and (4) cytoskeletal alteration. Below are some observations regarding these 17 up- and 38 down-expressed proteins based on their biological functions and properties:

7 upregulation and 10 downregulation proteins were directly involved in the four disease mechanisms mentioned above (Table 2). Some of these proteins serve multiple roles as immunological modulators, contributing to epidermal proliferation. Some proteins perform cytoskeletal function for maintaining epidermal proliferation. 60% up- and 40% downregulated proteins were differentiated into cellular proliferated proteins. mTOR, a protein contributing to epidermal proliferation, had the highest fold change among all observed proteins, which was seen to have significantly reduced in the treatment group. Decorin, an epidermal differentiation protein, was significantly elevated in ScF-treated rats with the highest fold change in the upregulated proteins followed by complement C3, lumican, serpinA3, serotransferrin, hemopexin, and HSPA2, respectively. Another downregulated protein was also categorized with its specific function such as telomerase activity (TEP1), a type of keratin (KRT5, KRT14, and KRT75), inflammatory regulator (S100A8, ANXA1, Lyz1, and BSPB1), and apoptotic indicator (ANXA5). The categorization of proteins on the basis of their mechanisms is shown in Fig. 4D. In addition, all of their functions are demonstrated in Table 2.

**Table 2:** The list of up- and downregulated proteins associated with psoriasis pathogenesis, in psoriasis-affected rats treated with ScF.

Protein alteration	Protein accession	Protein name	Function	Psoriasis pathogenesis	Coverage (%)	Protein score	Fold change
Upregulation	PGS2_RAT	Decorin	Decorin is a leucine-rich proteoglycan in associated with collagen fibrils and is known for its antifibrotic, anti-inflammatory, and anti-cancer effects^52^. A reduction in decorin might negatively affect the terminal differentiation, and consequently enhance epidermal proliferation in relation to the increment of TGF-β, TNF-α, and IL-1β^53^	1 and 3^#^	17.2	178	4.33
	CO3_RAT	Complement C3	Complement C3 plays a significant role in the activation of the complementary system,^+^ and is useful in predicting psoriatic arthritis and cardiometabolic risk in psoriasis^54^	1 and 3*	15	460	4.11
	LUM_RAT	Lumican	Lumican is an extracellular matrix proteoglycan involved in collagen fibrillogenesis, matrix turnover, and proliferation response^55^	3 and 4^#^	30.5	221	2.91
	SPA3N_RAT	Serine protease inhibitor A3N (SerpinA3)	Serpin A3 is an inhibitor of several proteases^+^. *SERPINA3* gene mutation or upregulation of serpinA3 is a predisposing factor of pustular psoriasis^56^	3*	18.2	134	2.90
	TRFE_RAT	SerotranScFerrin	SerotranScFerrin is an abundant blood plasma glycoprotein, which is upregulated in psoriatic skin, and is involved in cellular proliferation^57^	3*	48.9	1218	2.63
	HEMO_RAT	Hemopexin	Hemopexin is the plasma protein with the highest binding affinity to heme, which is elevated in respiratory diseases, particularly asthma and chronic obstructive pulmonary disease (COPD)^58^	1^#^	39.6	546	2.13
	HSP72_RAT	Heat shock-related 70 kDa protein 2 (HSPA2)	HSPA2 is a stress-induced chaperone, contributing to the early step of keratinocyte differentiation and maintenance of epidermal homeostasis^59^	3^#^	22.1	117	2.00
Down-regulation	FRAP_RAT	Serine/threonine-protein kinase mTOR (mTOR)	mTOR is a serine/threonine kinase, that regulates cell growth, autophagy, and the actin cytoskeleton in response to the stimuli and cytokines. The mTOR signaling proteins are upregulated in psoriatic skin and proliferation keratinocytes^20,22^	3*	19.3	120	22.50
	TEP1_RAT	Telomerase protein component 1 (TEP1)	TEP1 is a component of the ribonucleoprotein complex responsible for telomerase activity enhancement in psoriasis^60,61^	3*	14.5	106	6.00
	K2C75_RAT	Keratin, type II cytoskeletal 75 (KRT75)	KRT75 is an essential component of keratin intermediate filaments in hair^+^. Upregulation of KRT75 may be associated with atopic dermatitis and psoriasis via peroxisome proliferator-activated receptors^62^	1, 3, and 4*	13.3	279	3.20
	K1C14_RAT	Keratin, type I cytoskeletal 14 (KRT14)	Alteration of the KRT14 gene may play a possible role in the pathogenesis of psoriasis and its severity^32^	3 and 4*	50.7	932	2.80
	K2C5_RAT	Keratin, type II cytoskeletal 5 (KRT5)	KRT5 differential expression is more relevant to atopic dermatitis than in psoriasis^63^	3 and 4^^^	33.5	805	2.76
	S10A8_RAT	Protein S100-A8 (S100A8)	S100A8 is a calcium- and zinc-binding protein, which plays an important role in the regulation of inflammatory processes and immune responses^+^. S100A8-S100A9 are the most upregulated proteins expressed in the psoriatic skin^64^	1 and 2*	55.1	311	2.65
	ANXA1_RAT	Annexin A1 (ANXA1)	ANXA1 is a target for T cells- and can drive activated T cells toward Th17 helper T cells, thus plays a role in psoriatic arthritis^65^	1*	38.4	315	2.31
	LYSC1_RAT	Lysozyme C-1 (Lyz1)	Lyz1 is a lysozyme that has a bacteriolytic function^+^. Psoriatic skin lesions have high expression of lysozyme^66^	1*	65.5	378	2.27
	HSPB1_RAT	Heat shock protein beta-1 (BSPB1)	*HSPB1* is a small member of HSP20 responsible for the stress environment reaction^+^. High levels of *HSPB1* are observed in the keratinocytes in inflammatory skin diseases such as psoriasis^67^	3*	32	119	2.20
	ANXA5_RAT	Annexin A5	Annexin A5 is a cellular protein used for detecting apoptotic cells^+^. The induction of apoptosis leads to the regression of psoriatic hyperplasia, while the decrease in physiological apoptosis influences psoriatic hyperplasia^68^	2*	22.6	168	2.11

Psoriasis pathogeneses* in this table were categorized as follows: (1) inflammation, (2) apoptosis, (3) cell proliferation, and (4) cytoskeletal alteration.

^#^and ^^^High and low possibility to be related to psoriasis pathogenesis.

^+^A basic functional source of each protein is based on the UniProt database (www.uniprot.org) or the National Library of Medicine, National Center for Biotechnology Information, NIH, USA.

#### ScF alleviated keratinocyte proliferation via the mTOR pathway and modulated inflammation by IL-17 downregulation

**Figure 5:**
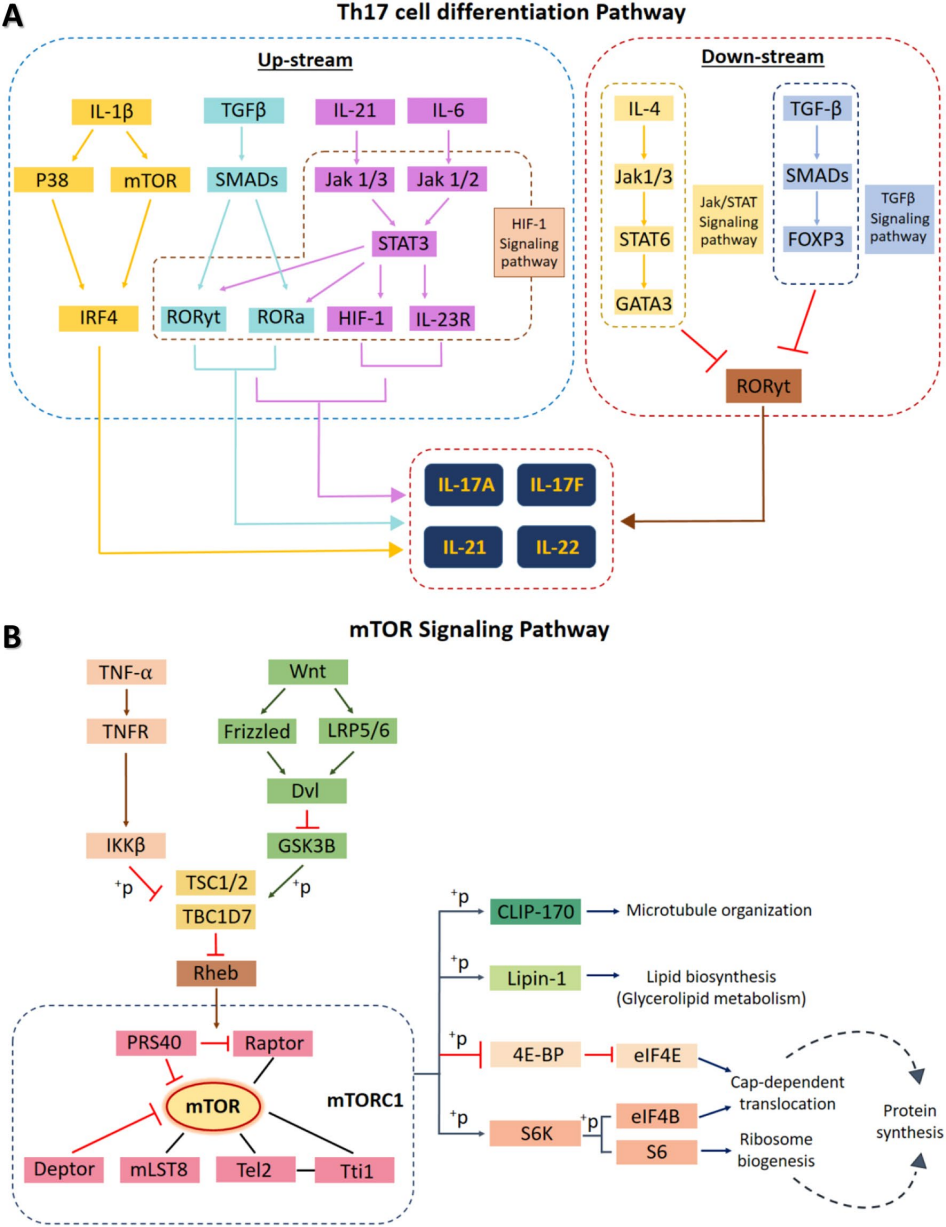
Main associated pathways in anti-psoriatic property of ScF: (**A**) Th17 cell differentiation pathway adapted from KEGG pathway analysis, (**B**) Th17 cell differentiation pathway adapted from KEGG pathway analysis.

According to the proteomic analysis and immunohistochemical study, there is a definite connection between (1) mTOR (mechanistic target of rapamycin) protein (Table 2), (2) the cytokine expressions of TNF-α, Wnt, IL-1β, TGF-β, IL-6, IL-17, and IL-21 (Fig. 3), and (3) psoriasis pathogenesis; particularly proliferation and inflammation in relation to Th17 cell differentiation and mTOR signaling pathways, as shown in Fig. 5. A KEGG pathway analysis indicates that ScF modulates inflammation by the downregulation of IL-17 production cascade response from the depletion of IL-1β and mTOR levels through the Th17 cell differentiation pathway. In addition, the decrease in TGF-β may lead to downregulation of IL-17 in ScF-treated rats. Unfortunately, IL-21 and IL-22 levels did not significantly reduce in the ScF treatment group. Nevertheless, downregulation of TNF-α and Wnt resulted in the inhibition of epidermal proliferation via the mTOR signaling pathway.

#### Additional anti-psoriatic mechanisms of ScF

Apart from the main influence of ScF on psoriasis treatment via the mTOR signaling and Th17 differentiation pathways, ScF also demonstrated therapeutic effects via another mechanism. An increment in decorin (Table 2) resulted in the inhibition of epidermal proliferation in association with the reduction of TGF-β, TNF-α and IL-1β expression (Fig. 3). A reduction in telomerase activity due to TEP1 downregulation led to an impediment in epidermal overgrowth. A depletion of cytoskeletal keratin proteins (KRT14 and KRT75) possibly alleviates the psoriasis severity. In addition, the decline of inflammatory regulators such as S100A8, ANXA1, Lyz1, and BSPB1 may possibly help to improve psoriatic skin conditions.

#### Chemico-protein interaction among amino acids of sericin and detected proteins

**Figure 6:**
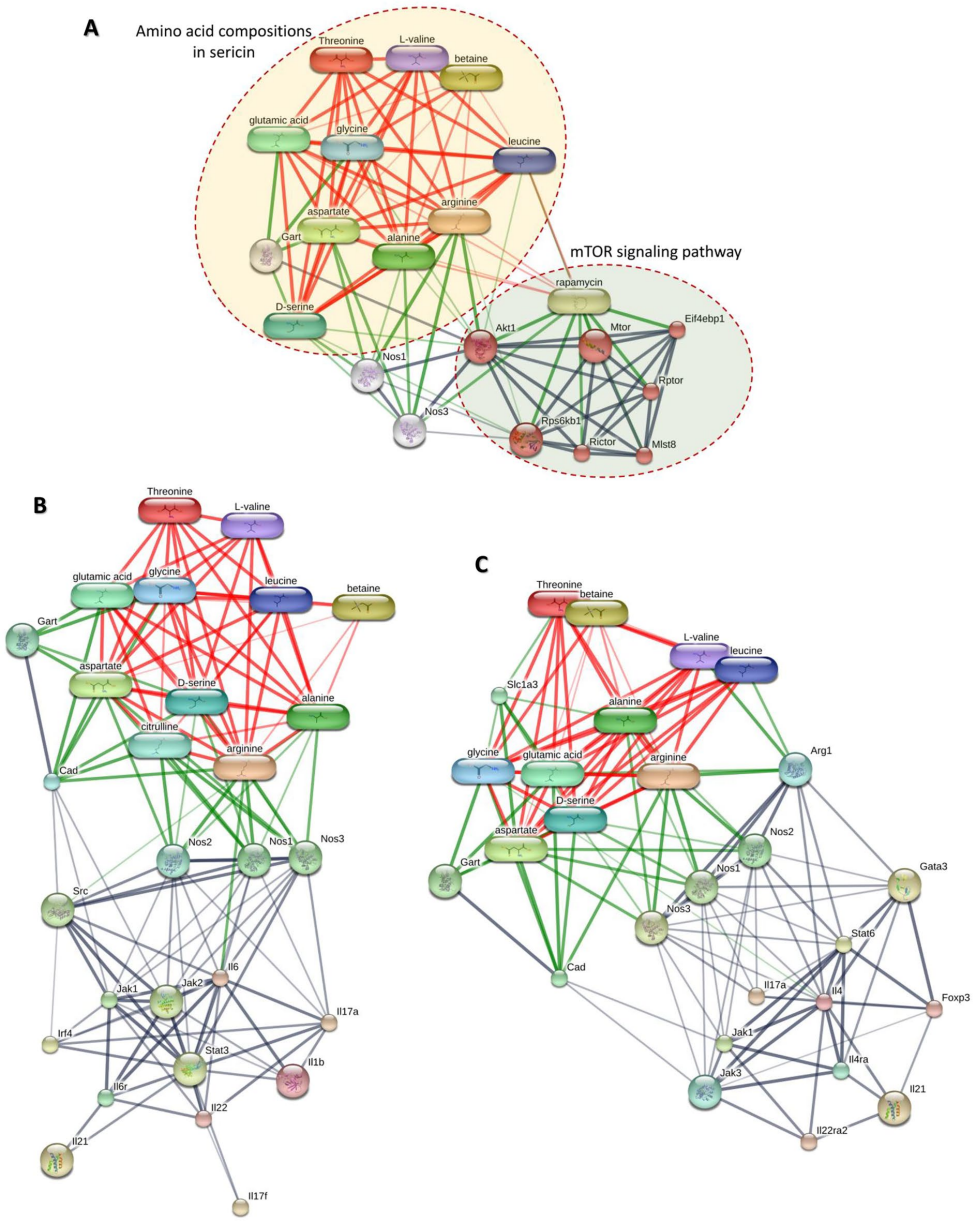
The chemical-protein interaction between the amino acid compositions in sericin and related pathways: (**A**) The interaction of sericin amino acids with the protein-involved in the mTOR pathway, (**B**) The interaction of sericin amino acids in up-streaming proteins-involved in the Th17 cell differentiation pathway, (**C**) The interaction of sericin amino acids in down-streaming protein-involved in the Th17 cell differentiation pathway. Red, green, and gray lines depict amino acid interaction, chemico-protein interaction, and protein interaction, respectively.

Chemico-protein interaction analysis demonstrates that some amino acids in sericin, such as serine, leucine, alanine, arginine, and glycine have interactions with proteins in the mTOR pathway (Fig. 6A). Arginine predominantly interacts with protein kinase B (Akt), whereas serine, alanine, leucine, and glycine have limited interactions with Akt and ribosome protein S6 kinase B1 (Rps6kb1). Arginine also sparingly reacts with Rps6kb1. In contrast to the Th17 differentiation pathway, there is no interaction between amino acids and proteins present in sericin, in these up- and downstream mechanisms (Fig. 6B,C).

## Discussion

In this study, we investigated the therapeutic and non-toxic properties of sericin-coated thin polymeric films (ScF) fabricated using photocrosslinked fibroin. This includes understanding its effects on disease progression and mechanisms in a psoriasis rat model based on several approaches. The use of the 2D fibroin substrates is important in terms of providing a mechanically robust, flexible, and compatible platform for the application of sericin. The films themselves are conformable to soft tissue and may be degraded under proteolytic conditions. We can safely postulate that the ScF relieves the severity of psoriasis by the reduction in epidermal cell overgrowth with anti-proliferative effect through the mTOR pathway through: (1) upregulation of anti-proliferative protein (decorin), (2) downregulation of antimicrobial peptide (β-defensin), apoptotic proteins (caspase-3 and -9), chemokine (CCL-20), proinflammatory cytokines (TNF-α, IL-1β, and IL-17), inflammatory modulator (S100a14, S100A8, ANXA1, Lyz1, and BSPB1), proliferative related proteins (TGF-β, Wnt, mTOR, and TEP1), and keratinized proteins (KRT14 and KRT75). According to these underlying mechanisms, the reduction in hyperkeratosis, epidermal edema and swelling, squamous cyst, and folliculitis were distinctly observed in ScF-treated rats compared with the non-treatment group (Fig. 2). A schematic for the mechanism involved in ScF-mediated psoriasis improvement is deposited in Supplementary Fig. S1 online. In addition, ScF demonstrated non-irritating quality (Fig. 1) and fairly maintained hematological values (Table 1).

It is important to note that the combination of sericin and fibroin exhibits good biocompatibility. The early idea that sericin induced inflammatory response was shown to be not the case by important studies over the years, which demonstrated that both fibroin and sericin are immunologically inert when separate^11^. Other blends of these proteins have been used to promote angiogenesis^12^ and form scaffolds for tissue engineering and drug delivery^13,14^. Our work further confirms that physical combination of these two proteins provides negligible inflammatory response, with a therapeutic benefit. The present study provides a prototype of an adjunctive therapy for psoriasis treatment, applying photocrosslinked silk fibroin films, which provide unique tunability in terms of thickness, mechanical strength and biodegradation. While chemically crosslinked silk fibroin films may also be used, the use of photocrosslinkable silk provides advantages in terms of potentially targeted surface treatments. Use of photolithographic methods for micropatterned fabrication of 2D and 3D structures are promising as functional biointerfaces, flexible microdevices and wearable sensors, due to precisely controlled properties and scalable production^8–10,15,16^. However, their applications in treatment of skin conditions are still limited. This work shows the benefits of combination of photocrosslinked fibroin sheets and sericin extraction for psoriasis treatment in this in vivo study. In future, surfaces of the ScF can be precisely patterned to provide for targeted spatial and temporal application of sericin^7^. Pattern development, formula setting and evaluation, and cost effectiveness would also need further study.

According to the proteomics study, the highest fold-change protein (Table 2) observed in the downregulated component was the mTOR protein. The mTOR signaling cascade in psoriasis has been well recognized for decades^17–22^. It plays a pivotal role in the regulation of cellular-proliferation, -survival, and -motility in association with energy and nutrient availabilities. mTOR pathway dysregulation leads to uncontrolled epidermal-proliferation and -inflammation, especially in psoriasis, atopic dermatitis, pemphigus, acne, cutaneous T cell lymphoma, and melanoma. Inflammatory cytokines, (TNF-α, IL-1β, IL-17A, IL-21, and IL-22), induce aberrant mTOR activity, leading to epidermal cell overgrowth and abnormal epidermal organization. In agreement with our study, psoriatic skin-treated with ScF had a significantly low levels of mTOR protein alongside the downregulation of TNF-α, IL-1β, and IL-17 (Fig. 3) indicating an inhibitory effect of ScF on mTOR signaling (Fig. 5) to mitigate psoriasis severity. In addition, the mTOR signaling pathway is a part of the downstream loop of the phosphatidylinositol 3-kinase (PI3-K)/protein kinase B (Akt) pathway, which contributes to cellular growth, proliferation, and metabolism^23^. Hence, considering the regulation of proliferation and cellular growth, the inhibition of PI3-K/Akt and mTOR signaling could be a promising therapeutic strategy in psoriasis such as the Vitamin D analog 1α, 25-dihydroxyvitamin D3-3-bromoacetate^24^, double kinase inhibitor^22^, as well as ScF. ScF exhibited some collateral inhibition pathways in the retardation of epidermal proliferation and inflammation, both up- and down-streams: decorin, TEP1, KRT14, KRT75, S100A8, ANXA1, Lyz1, and BSPB1. All of these proteins function involved with psoriasis pathogenesis are mentioned in Table 2.

Studies on the proliferative and other related effects of sericin based on PI3-K/Akt and mTOR signaling have not been much established. It was earlier reported that sericin inhibits breast cancer cell (triple negative breast cancer; TNBC) proliferation via the PI3-K/Akt pathway, by inducing cell cycle arrest and promoting cellular apoptosis^25^. The enhancement capacity of sericin on glucose transport and liver glycogen synthesis was shown via the insulin-PI3-K/Akt pathway^26^. Apart from PI3-K/Akt and mTOR signaling, sericin is also involved in another pathway that actively influences wound healing, namely the MAPK/ERK (mitogen-activated protein kinases/extracellular signal-regulated kinase) pathway for corneal resolution^27^. This pathway is well known as the main signaling pathway for proliferation, similar to PI3-K/Akt and the mTOR signaling pathway.

Th17-derived proinflammatory cytokines (IL-17A, IL-17F, IL-21, IL-22, and IL-26) have a critical role in the pathogenesis of several autoimmune diseases such as psoriasis, multiple sclerosis, rheumatoid arthritis, and inflammatory bowel disease^28^. IL-17 promotes the *S100A8* and *S100A9* gene expression as an alarm signal during the inflammatory response of keratinocytes in psoriasis^29^. Our previous study demonstrated that a cream-based sericin formulation reduces the severity of psoriasis through Th17 cells by interfering with the Janus kinase (JAK)-signal transducer and the activator of transcription (STAT) pathway^5^. Sericin cream also modulates epidermal immunity via galectin-3 (*LGALS3*) and sphingosine-1-phosphate lysate1 (*SGPl1*), and epidermal proliferation via nucleoside diphosphate kinase B (Nme2). In the present study, the connection between an upregulation of mTOR protein (from proteomics approach) and the reduction in IL-1β, IL-17, and TGF-β (from immunohistochemistry) was observed, which is most relevant to the Th17 cell differentiation pathway (Table 2 and Fig. 5). In consonance with our previous study, there were some overlapping mechanisms that share the effect of sericin on psoriasis pathogenesis, particularly the Th17 cells, which can be the focal point for psoriasis improvement. However, some different pathways might be involved in the preparation form used in the studies. In addition, IL-8 also plays an important role in the pathogenesis of psoriasis especially at local level in psoriatic plaque area as characterized by micro-abscesses or pustule in which a number of epidermal neutrophils were deposited^30,31^. In the present study, an epidermal IL-8 expression could not be reduced in ScF treatment when compared to other groups. This might be related to the high occurrence of pustule formation in ScF-treated rats (Fig. 2C), since neutrophils secrete IL-8.

The histopathological study investigated the occurrence of typical lesions in an imiquimod-induced psoriasis rat model, particularly hyperkeratosis, acanthosis, dermatitis, and folliculitis. An additional mechanism besides the above-mentioned pathway—alteration of keratinized proteins, was also involved in the formation of hyperproliferative lesions in psoriasis. Keratin mutations, epidermal- and hyperproliferative-keratin mutations, are the main causative factors for psoriasis, leading to acanthosis and abnormal terminal differentiation, and contributing to disease severity and relapse of psoriatic condition, respectively^32^. Apart from the common lesions in the psoriasis rat model, epidermal edema or swelling, and squamous cysts were also observed. Epidermal edema or spongiosis, with widening of the intercellular space, is a common feature of skin inflammation, in association with the rupture of the desmosome and formation of intraepidermal vesicles^33^. Squamous cysts most likely arise from injured pilosebaceous units, in which squamous epithelial cells producing keratin are trapped in the dermis^33^. Our results highlight the ability of ScF in the reduction of acanthosis, hyperkeratosis, and squamous cysts when compared with the calcitriol- and non-treatment groups (Fig. 2). However, dermatitis and pustules were not alleviated as much, probably owing to the period of treatment.

Hematological changes such as anemia, thrombocytosis, neutrophilia, eosinophilia, and monocytosis have been claimed to be associated with psoriasis for several decades^34–40^. These alterations are important complications and can aggravate disease severity. For instance, Erythrodermic psoriasis is a rare life-threatening subtype of psoriasis accompanied by anemia and thrombocytosis^36^. In addition, polymorphonuclear cell- and monocyte-platelet aggregate formation may be a promoting factor for psoriasis pathology and disease severity^34,40^. In agreement with our study, the imiquimod-induced psoriasis rat model markedly demonstrated leukocytosis, eosinophilia, basophilia, and neutrophilia (Table 1). Mild anemia and thrombocytosis were also observed. However, monocyte depletion was observed. The results suggested that like standard treatments, ScF significantly reduced the recruitment of neutrophils. Further, liver and kidney functional enzymes also remain fairly preserved under ScF treatment, reflecting its safety. In contrast, calcitriol treatment elevated the levels of SGPT and SGOT, compared to ScF- and non-treatment groups, indicating a primary disadvantage of chemical-based treatment in psoriasis.

Chemico-protein interactions indicated the connections between the amino acid composition in sericin extraction to proteins in the mTOR pathway (Fig. 6). In the present study, the main interaction was found between arginine and Akt, a key cellular process including cell proliferation, apoptosis, and metabolism. Prior work has shown that Akt is activated by prolonged deprivation of arginine, and that leucine leads to reactivation of mTOR signaling^41^. Moreover, alanine uptake may also inhibit Akt by completely reversed leukotriene D4 (LT)-mediated inhibition of Na^+^-Dependent Alanine Cotransporter (ASCT1)^42^. In agreement with our study, the enrichment of arginine, leucine, and alanine, which are the components of sericin, could inhibit mTOR signaling, as demonstrated by a downregulation of mTOR protein in ScF-treated rats.

In summary, we propose that ScF—a combination of sericin extraction and 2D flexible, photocrosslinked fibroin films, inhibits the mTOR pathway and Th17 differentiation signaling to promote psoriasis resolution. The underlying mechanisms contributed to several factors: proliferative related proteins (TGF-β, Wnt, mTOR, TEP1, and decorin), antimicrobial peptide (β-defensin), apoptotic proteins (caspase-3 and -9), chemokine (CCL-20), proinflammatory cytokines (TNF-α, IL-1β, and IL-17), inflammatory modulator (S100a14, S100A8, ANXA1, Lyz1, and BSPB1), and keratinized proteins (KRT14 and KRT75). Therefore, ScF provides a potentially promising modality for psoriasis treatment due to the favorable properties of sericin in terms of anti-psoriatic effects and the stimulation of healing.

## Materials and methods

### Preparation of sericin-coated thin polymeric film fabricated from fibroin (ScF)

Preparation methods for each component used to formulate the dressing material in this study are mentioned below.

#### Photo-crosslinkable fibroin synthesis

The fibroin films used in this study are based on photocrosslinkable fibroin (photofibroin) which provides unique tunability in terms of mechanical properties as well as surface morphology. The photofibroin was prepared according to the procedure described by Xu et al.^7^. In brief, pure silk fibroin obtained from *Bombyx mori* cocoons after a standard extraction protocol^43^ was dissolved in 1 M LiCl/DMSO (lithium chloride/dimethyl sulfoxide). It was then treated with 2-isocyanatoethyl methacrylate (IEM) for 5 h at 60 °C, maintaining a constant flow of nitrogen. The solution was then added to cold ethanol, to precipitate out the methacrylated protein^44^. The product was washed with a 1:1 ratio of cold ethanol and acetone, centrifuged, and lyophilized for 24 h.

#### Fabrication of flexible fibroin films

The lyophilized photofibroin powder was dissolved in 7.5% w/v formic acid (Acros Organics 98%) and 2.5% w/v of photoinitiator (Irgacure2959, BASF). The solution was poured for casting on plain glass slides, evaporated by air drying for 20 min, crosslinked by exposure to a 365 nm UV lamp (Lumen Dynamics OmniCure 1000 system) for 3 s at 20 mW cm^−2^, and was then dipped in deionized (DI) water to obtain a crosslinked fibroin film. The films were kept in a desiccator until use. Films were typically 25–40 µm in thickness. Films were cut into identical 1.0 cm × 1.0 cm pieces and soaked in 5% sericin solution^5^ for 24 h at room temperature (Fig. 1Ai). Films were then exposed to ultraviolet (UV) radiation, before being used in further experiments that included bioimplantation and psoriasis studies. To differentiate from silk fibroin (SF), in this work, we refer to the sericin-coated thin photofibroin films as ScF.

#### Sericin extraction

Sericin extraction was performed by an autoclaving method^45^. Briefly, *Bombyx mori* cocoon shells, received from Chul Thai Silk Co. Ltd., Phetchabun Province, Thailand, were autoclaved in distilled water for 1 h at 120 °C. Dissolved sericin was filtered and stored in a desiccator until use. Amino acid composition was examined by the Central Laboratory (Thailand) Co., Ltd., Bangkok, Thailand.

### Ethical statement

Animal studies were approved by The Faculty of Tropical Medicine ACUC, Mahidol University (Approval No. FTM-ACUC 023/2021). Animal experimental protocols were in accordance with the ARRIVE guidelines 2.0, the National Research Council of Thailand's Guidelines for the Use of Animals and the Thai Animals for Scientific Purposes Act, formulated in B.E. 2558. Forty 8-week-old female Wistar rats were obtained from the Nomura-Siam International company, Thailand. All rats were housed in an environment controlled-room with temperature maintained at 25 ± 2 °C, 65 ± 10% humidity, 12-h/12-h light/darkness cycles; and they were provided access to a standard diet and filtrated water at all times.

### Bioimplantation study

#### Experimental protocol

To investigate sericin-coated photofibroin film (ScF) interactions in terms of host inflammatory response, a bioimplantation study was conducted according to the ISO 10993-6:2016 guideline as mentioned in our previous study^46^. Absorbable Chromic catgut No. 2-0 was used as a control material due to its appropriate biocompatibility and common use in surgery. Twenty-five rats were used in this study. They were randomly divided into five equal groups with differently timed endpoints (3-, 7-, 14-, 21-, and 28-days post-implantation). Following anesthesia with 50 mg/kg of thiopentone intraperitoneally, they were shaved along the dorsal midline of the thoracolumbar area. The surgical site was scrubbed with betadine and a 2 cm incision was made. 1 cm × 1 cm of ScF and 1 cm of Chromic catgut were inserted into the blunt socket of both sides. The epidermal incision was closed with Vicryl No. 4-0 sutures. All rats received 15 mg/kg of tramadol, intramuscularly as an analgesic for 3 days post-surgery. The wound was carefully observed daily until suture removal on day seven post-surgery.

#### Sample collection

At the endpoint for each group, all rats were humanely euthanized by an overdose of carbon dioxide inhalation. All implanted materials were excised with some adjacent tissues. They were preserved in 10% neutral-buffered formalin (NBF) for histopathological study and 2.5% glutaraldehyde in sucrose phosphate buffer (SPB) for electron microscopy.

#### Histopathological study

To assess inflammatory or irritation levels induced by ScF and its control, a histopathological study was carried out. Fixed specimens were processed as per standard protocol for tissue processing, and were subjected to dehydration with ethanol, infiltration with xylene, embedding with paraffin, cutting into 5 µm sections, and staining with hematoxylin and eosin (H&E). The sections were examined under a light microscope (BX41, Olympus, Japan) with a focus on cellular infiltrations (neutrophils, lymphocytes, macrophages, plasma cells, and giant cells) and histopathological changes (necrosis, neovascularization, fibrosis, and fat infiltration) in the tissue interfacing area. All these criteria were observed and graded as follows: 0: absent, 1: rare, 2: mild, 3: moderate or advanced stage, and 4: severe or end stage. The final score was calculated by the subtraction between the total score of the control material and ScF.

#### Electron microscopic study

To characterize the ultrastructure of ScF both before and after implantation, scanning electron microscopy (SEM) was performed. Specimens were fixed with 1% osmium tetroxide, dehydrated with ethanol, dried in a critical point drying device (CPD300 auto, Leica, Wetzlar, Germany), coated by a coat-sputter (Q150R PLUS, Quorum, East Sussex, England), and examined under an SEM (JSM-6610LV, JEOL, Tokyo, Japan) with emphasis on the material alteration at the surface.

### Imiquimod-induced psoriasis rat model

Imiquimod-induced psoriasis rat model was used for the evaluation of potential curative and toxic effects of ScF in connection with disease progression and resolution. Fifteen rats were included in this study. They were shaved along the dorsal midline in the thoracic area. Based on our recent studies^5,6,47^, 62.5 mg of imiquimod was applied daily on the experimental site measuring 1.5 cm × 1.5 cm for seven days, which was considered as an induction period. Macroscopic skin lesions such as epidermal scales, redness, and thickening were monitored. All rats were randomly divided into three equal groups with different treatments (ScF test material, standard treatment; 3 µg/g calcitriol ointment, and non-treatment; paraffin) and were treated with the appropriate treatment for 7 days, which was the treatment period. The experimental site was wrapped with a Fixomull stretch to protect the test site. To maintain psoriatic skin conditions during the treatment period, imiquimod was continuously administered to the experimental site after the induction period.

#### Sample collection

Seven days post-treatment, all rats were humanely euthanized by carbon dioxide inhalation. Exsanguinated blood collection was performed by a cardiac puncture. The blood samples were sent for hematological and blood clinical chemistry evaluation, performed by the National Laboratory Animal Center, Mahidol University. Skin experimental sites were removed, separated into three parts, and preserved (1) in 10% NBF for histopathological and immunohistochemical studies, (2) in RNA*later* stabilization solution for molecular analysis with RT-qPCR, and (3) in − 80 °C freezer for proteomics study.

#### Histopathological study

Fixed skins were subjected to standard tissue processing. The skin sections were examined under a light microscope focusing on psoriasis-related epidermal lesions, particularly hyperkeratosis, acanthosis, pustules, epidermal edema and cysts, folliculitis, and dermatitis. These lesions were scored using H-score (0–300; multiplication of a severity score [0–3; 0–absent, 1–mild, 2–moderate, and 3–severe] and an extent of distribution [0–100%]). Moreover, epidermal thickening was measured by image analysis using ImageJ software, version 1.36 (NIH, USA). Colored images were acquired using a digital camera (DP20, Olympus®, Japan) at 400× magnification. Epidermal length was examined from stratum basale to stratum corneum by drawing a straight line and measuring the distance in µm unit (5 measurements/section).

#### Immunohistochemical study

To characterize the expression of some markers involved with psoriasis pathogenesis, immunohistochemical staining was performed. Rabbit isotype polyclonal antibodies (MyBioSource, USA) were used, which measured the levels of: (1) Psoriasis-related protein: β-defensin (antimicrobial peptide), (2) Apoptosis: Caspase-3 and -9, (3) Proinflammatory cytokines: Tumor necrotic factor (TNF)-α, interleukins (IL)-1β, -6, -8, -17, -21, and -22, (4) Inflammatory chemokine: C–C motif chemokine ligand 20 (CCL20), (5) Anti-inflammatory cytokine: Transforming growth factor (TGF)-β, (6) Antioxidation: Nuclear factor erythroid 2-related factor 2 (Nrf-2), (7) Proliferation protein: Wingless-related integration site (Wnt). Sections were further subjected to microwave-induced antigen retrieval in citrate buffer (pH 6) following deparaffinization with xylene and hydration with ethanol. For blocking the endogenous peroxidase activity and non-specific binding, sections were treated with 1% v/v of hydrogen peroxide in methanol and 2% v/v of bovine serum albumin (BSA; [EMS, USA]) respectively. Sections were incubated with each primary antibody, polymer HRP anti-mouse/rabbit labeling (DAKO, Denmark), diaminobenzidine visualization (DAKO, Denmark), and then counter-stained with hematoxylin. Finally, immunolocalization was examined under a light microscope.

The level of expression of each protein was measured in terms of the H-score (percentage area of expression × intensity score). ImageJ software was used to quantify the immuno-distribution area in terms of percentage. Color images (10 images/group) were captured at 400× magnification. The immunolabeled area was measured by a threshold mode to obtain the percentage of positive pixels after the conversion of images to grayscale. Intensity was scored from 0 to 3 and was classified into four grading scales: 0–negative staining, 1–low-intensity staining, 2–moderate-intensity staining, and 3–high-intensity staining.

#### Quantitative real-time polymerase chain reaction (RT-qPCR)

To measure the expression of genes involved in psoriasis pathogenesis, mRNA expression levels of *FLG*, *caspase-14*, *S100a7a*, and *S100a14* genes were measured by RT-qPCR.

##### RNA extraction

An RNA purification kit (RNeasy Mini Kit, Qiagen, Canada) was used to extract mRNA from the rat skin samples preserved in the RNA*later* solution. Briefly, samples were homogenized in a lysis buffer and loaded in a spin column. After RNA bound to the column, it was washed with the buffer. RNA was eluted in RNase-free water, RNA concentrations were measured on NanoDrop™ 2000/2000c spectrophotometer (Thermo Scientific, USA).

##### RT-qPCR

RT-qPCR was performed by using the iTaq Universal SYBR Green Supermix (Biorad, USA). In the CFX96 Touch™ Real-time PCR detector, the primer pairs were used as indicated; FLG: F-5′AGATGTGGACCACGATGACAA3′, R-5′TAGTGCTGGATCCTCGTCTTTT3′, β-Actin: F-5′CACTATCGGCAATGAGCGGTTCC3′, R-5′AGCACTGTGTTGGCATAGAGGTC3′, Caspase-14: F-5′CAGACCCTGACGGATGTGTTC3′, R-5′GCGAGGGTGCTTTGGATTTCGG3′, S100a7a: 5′TAGTGTGCCTCGCTTCATGGAC3′, R-5′CACAACTGCCGGTGAAACTGA3′, and S100a14: F-5′AACAATGGGACAGTGTCGGTC3′, R-5′ACTGCTGGGTAACCAGGTCTC3′ for amplification and denaturation (Bio-Rad, Germany). Using 2^**−**∆∆Ct^ method, the individual gene expression levels were calculated. The expression levels of *β-Actin* were used as a reference for accurate normalization of gene expression data.

#### Proteomics study

##### Sample preparation and protein extraction

Pool samples of psoriatic skins from rats treated with or without ScF were subjected to proteomics study. The samples (30 mg/group) were ice cool ground in 300 μl of lysis buffer (1% SDS, 0.5% NaCl, 1% Triton-X) in an ultrasonicator, and centrifuged at 12,000 rpm, 10 min at 4 °C. The supernatant solution was collected for the next step of proteomics analysis.

##### Gel electrophoresis for protein separation

Prior to gel electrophoresis, the total proteins in the supernatant solution were measured by Bradford protein assay (Bio-Rad®, USA) using a spectrophotometer (Thermo Scientific, USA). 30 μg of sample in each group was loaded into the sodium dodecyl sulfate–polyacrylamide gel electrophoresis (SDS-PAGE) device, using 4% stacking and 12% separating gels. Gel electrophoresis was performed with a constant voltage of120 V and current of 400 mA for 80 min (Bio-Rad®, USA). The gel was then stained with Coomassie Blue-R (CBB-R) for 10 min, and then was subjected to destaining by soaking in 30% methanol and 10% acetic acid for 2 h. The gel was washed in distilled water for 15 min and photographed by gel documentary (Bio-Rad®, USA). Each lane of the gel was cut into 13 pieces, and each piece of gel was placed in a new 1.5 ml tube.

##### In-gel tryptic digestion

The gel pieces were dehydrated with 50% acetonitrile (Merck®, USA). Protein reduction was performed by adding 7 mM DTT (Dithiothreitol) in 50 mM ammonium bicarbonate for 15 min at 60 °C. Iodoacetamide 250 mM was added to the samples for alkylation and incubated for 30 min in the dark at room temperature. The alkylation reaction was quenched by using 4 mM DTT in 50 mM ammonium bicarbonate and the solution was discarded. The gel was dehydrated by adding 100% acetonitrile and dried for 1 h at room temperature. For the tryptic digestion of protein, solution of trypsin in 50 mM ammonium bicarbonate was added to gel samples and incubated for 24 h at 37 °C. The peptides were added to acetonitrile and kept for 20 min. The peptide solutions were transferred in 1.5 ml tubes. All peptide samples were dried at 40 °C by CentriVap Vacuum Concentrators (Labconco, USA).

##### Protein Identification in rat psoriatic skin

The dried peptides were added to a solution of 0.1% formic acid for dissolution of the peptides, before being tested on a mass spectrometer. The samples were subjected to liquid chromatography in UltiMate® 3000 Nano-LC systems (Dionex, UK). The peptides were separated using an Acclaim PepMap RSLC C18 column, measuring 75 mm × 15 cm, at a flow rate of 300 nl/min. The mobile phase was composed of solutions A and B a mixture of 0.1% formic acid and 80% acetonitrile in 0.1% formic acid, respectively. The peptide solution was subjected to gradient elution using solution B from 4 to 50% for 30 min. The eluted peptides were identified using the peptide spectra using positive electrospray ionization system coupled with microTOF-Q II (Bruker, Germany). The MS and MS/MS spectra covered the mass range of m/z 400–2000 and 50–1500, respectively. Afterwards, the proteins were identified and quantified using Mascot Daemon version 2.3.02 (Matrix Science, UK). The protein database was acquired from SwissProt specific to *Rattus norvegicus* (search date 02 September 2022). For the search parameter settings, the methionine oxidation was a fixed modification and carbamidomethylation of cysteine was a variable modification. The exponentially Modified Protein Abundance Index (emPAI) value was used for semiquantitative determination of protein expression levels^48^. The fold changes in protein expression were reported by comparison of the emPAI between sericin film and non-treatment groups. The proteins that had a fold change in protein expression ≥ 2 were classified on the basis of the biological functions using the UniProt database (www.uniprot.org). The associated proteins in the pathogenesis and physiology of psoriasis were explored with respect to the biological pathways using the Kyoto Encyclopedia of Genes and Genomes (KEGG)^49–51^. The amino acids in sericin and associated protein interaction and protein–protein interactions were investigated via STITCH version 5.0 (European Molecular Biology Laboratory, Germany) (www.stitch.embl.de/).

### Statistical analysis

Statistical analysis was performed by GraphPad Prism, version 6.05. Significant levels were considered as *p* < 0.05 (*), < 0.01 (**), < 0.001 (***), and < 0.0001 (****). The data were calculated and expressed by mean ± standard error (SD) with the appropriate parametric and non-parametric independent *t*-tests or analysis of variance (ANOVA).

## Supplementary Information

Supplementary Figure S1.

Supplementary Figure S2.

Supplementary Table S3.

Supplementary Table S4.

## Data Availability

The data sets used in the current study may be shared upon a reasonable request to Sumate Ampawong, Ph.D.
