# Supplementary material for: Sericin coated thin polymeric films reduce keratinocyte proliferation via the mTOR pathway and epidermal inflammation through IL17 signaling in psoriasis rat model
Source: Sci Rep. 2023 Jul 26;13:12133. doi: 10.1038/s41598-023-39218-y (PMC10372088; doi:10.1038/s41598-023-39218-y)
Supplement: Supplementary file 1 — Supplementary Figure S1. [file 41598_2023_39218_MOESM1_ESM.pdf]

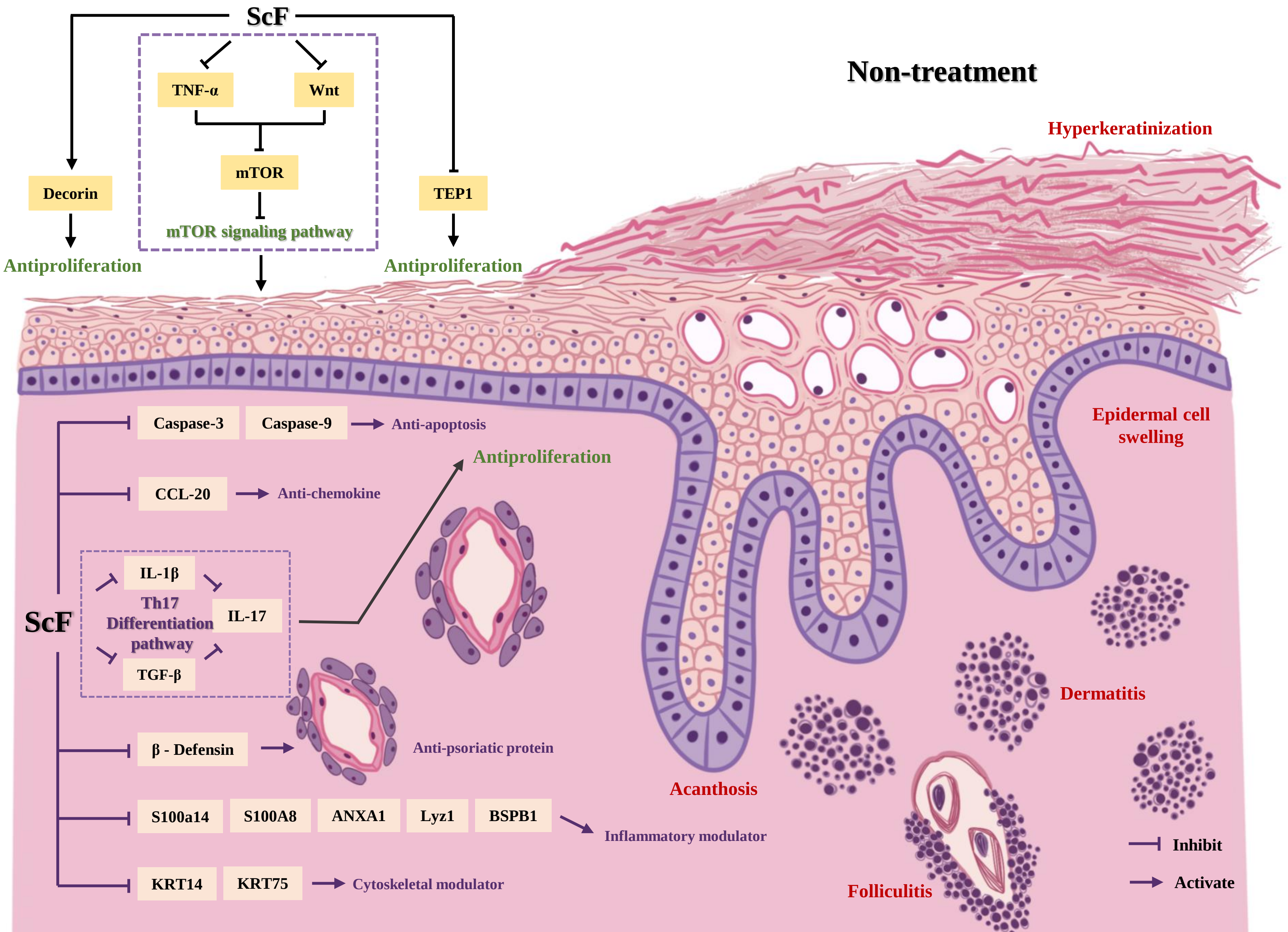

Figure S1: **A schematic for the mechanism involved in ScF-mediated psoriasis improvement**: including mTOR signaling pathway, Th17 differentiation pathway, anti-apoptosis, anti-chemokine, anti-psoriatic proteins, inflammatory modulator, and cytoskeletal modulator
