# Supplementary material for: Sericin coated thin polymeric films reduce keratinocyte proliferation via the mTOR pathway and epidermal inflammation through IL17 signaling in psoriasis rat model
Source: Sci Rep. 2023 Jul 26;13:12133. doi: 10.1038/s41598-023-39218-y (PMC10372088; doi:10.1038/s41598-023-39218-y)
Supplement: Supplementary file 2 — Supplementary Figure S2. [file 41598_2023_39218_MOESM2_ESM.pdf]

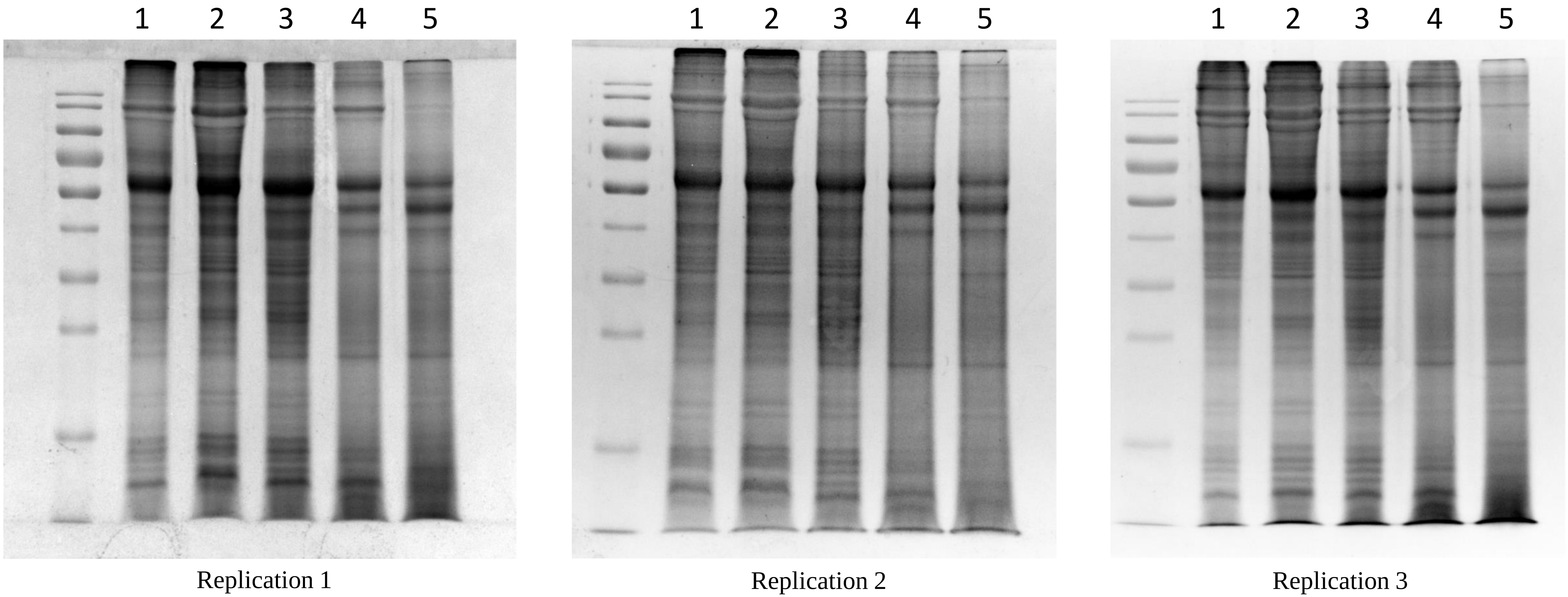

Figure S2: Full-length SDS-PAGE gels of psoriatic skin: The 1st lane to the 5th lane of each gel represented for non-treatment (using parafilm), pure hydrogel, sericin-based hydrogel, sericin- and curcumin-based hydrogel, and ScF treatment groups, respectively. In this study, the 1st lane and the 5th lane of each gel were cropped and arranged to the Figure 4B as a triplicate running.
