# Supplementary material for: Sericin coated thin polymeric films reduce keratinocyte proliferation via the mTOR pathway and epidermal inflammation through IL17 signaling in psoriasis rat model
Source: Sci Rep. 2023 Jul 26;13:12133. doi: 10.1038/s41598-023-39218-y (PMC10372088; doi:10.1038/s41598-023-39218-y)
Supplement: Supplementary file 3 — Supplementary Table S3. [file 41598_2023_39218_MOESM3_ESM.pdf]

Table S3. The 157 significant alteration proteins from 1,269 co-observational proteins divided by up-regulation.

Protein accession	Protein name	Fold change	p-value
H2A4_RAT	Histone H2A type 4	10	0.004738
PGS2_RAT	Decorin	4.333333333	0.000341035
CO3_RAT	Complement C3	4.111111111	0.000275365
LMNA_RAT	Lamin-A	3.5	0.008154672
A1BG_RAT	Alpha-1B-glycoprotein	3.307692308	0.001640713
A1AT_RAT	Alpha-1-antiproteinase	3.133333333	0.006772844
MDHC_RAT	Malate dehydrogenase, cytoplasmic	2.925925926	0.000731377
LUM_RAT	Lumican	2.913580247	0.001327189
SPA3N_RAT	Serine protease inhibitor A3N	2.904761905	0.000870061
PGK1_RAT	Phosphoglycerate kinase 1	2.785714286	0.002952805
HBB2_RAT	Hemoglobin subunit beta-2	2.662576687	0.006562916
TRFE_RAT	Serotransferrin	2.630630631	0.000918597
PYGM_RAT	Glycogen phosphorylase, muscle form	2.333333333	0.008760692
A1M_RAT	Alpha-1-macroglobulin	2.222222222	0.001155084
HEMO_RAT	Hemopexin	2.130434783	0.001931977
HBB1_RAT	Hemoglobin subunit beta-1	2.094117647	0.002681579
HSP72_RAT	Heat shock-related 70 kDa protein 2	2	0.009858252
K1C10_RAT	Keratin, type I cytoskeletal 10	1.733333333	0.003607441
TPM4_RAT	Tropomyosin alpha-4 chain	1.722222222	0.0025809
APOA1_RAT	Apolipoprotein A-I	1.68627451	0.003154238
HSP7C_RAT	Heat shock cognate 71 kDa protein	1.666666667	0.006381825
MCPT1_RAT	Mast cell protease 1	1.641975309	0.003558385
CO9_RAT	Complement component C9	1.6	0.009328321
K2C6A_RAT	Keratin, type II cytoskeletal 6A	1.571788413	1.184E-06
H31_RAT	Histone H3.1	1.571428571	0.003223036
PPARG_RAT	Peroxisome proliferator-activated receptor gamma	1.5	0.002052374
TPM1_RAT	Tropomyosin alpha-1 chain	1.489361702	0.002799989
H4_RAT	Histone H4	1.41875	0.002877408
H1T_RAT	Histone H1t	1.4	0.003371031
K2C73_RAT	Keratin, type II cytoskeletal 73	1.375	0.007756567
K1C42_RAT	Keratin, type I cytoskeletal 42	1.357142857	0.003379619
FETUA_RAT	Alpha-2-HS-glycoprotein	1.333333333	0.002906459
K1C13_RAT	Keratin, type I cytoskeletal 13	1.333333333	0.003775264
KPYM_RAT	Pyruvate kinase isozymes M1/M2	1.315789474	7.53773E-05
ALBU_RAT	Serum albumin	1.175680452	0.003086273
LDHA_RAT	L-lactate dehydrogenase A chain	1.170454545	0.009429266
K1C15_RAT	Keratin, type I cytoskeletal 15	1.136986301	0.001121454
DYN3_RAT	Dynamin-3	1.125	0.007446465
K2C1B_RAT	Keratin, type II cytoskeletal 1b	1.111111111	0.005567882
KNT1_RAT	T-kininogen 1	1.074074074	0.004169749
TPIS_RAT	Triosephosphate isomerase	1.066193853	0.003675832
TPM3_RAT	Tropomyosin alpha-3 chain	1.027777778	0.005000702
HBA_RAT	Hemoglobin subunit alpha-1/2	1.026190476	0.004581799
H12_RAT	Histone H1.2	1.018867925	0.004130336

Protein accession	Protein name	Fold change	p-value
APOH_RAT	Beta-2-glycoprotein 1	1	0.004992832
DDX52_RAT	Probable ATP-dependent RNA helicase DDX52	1	0.002106268
ECHD3_RAT	Enoyl-CoA hydratase domain-containing protein 3, mitochondrial	1	0.005000915
ATP5H_RAT	ATP synthase subunit d, mitochondrial	1	0.003436997
BCAR1_RAT	Breast cancer anti-estrogen resistance protein 1	1	0.007270457
CASQ1_RAT	Calsequestrin-1 (Fragment)	1	0.008427163
CBR1_RAT	Carbonyl reductase [NADPH] 1	1	0.008450625
CRP_RAT	C-reactive protein	1	0.004566037
CYTA_RAT	Cystatin-A	1	0.009967723
DESM_RAT	Desmin	1	0.006837389
EF2_RAT	Elongation factor 2	1	0.002156517
FABP5_RAT	Fatty acid-binding protein, epidermal	1	0.004910671
FABPH_RAT	Fatty acid-binding protein, heart	1	0.007165056
FGFR4_RAT	Fibroblast growth factor receptor 4	1	0.005152272
FINC_RAT	Fibronectin	1	0.006957035
FUBP1_RAT	Far upstream element-binding protein 1	1	0.002904278
GNAS1_RAT	Guanine nucleotide-binding protein G(s) subunit alpha isoforms XLas	1	0.008964533
ITAD_RAT	Integrin alpha-D	1	0.005078197
K1C12_RAT	Keratin, type I cytoskeletal 12	1	0.005109198
K1C18_RAT	Keratin, type I cytoskeletal 18	1	0.00573286
K1C24_RAT	Keratin, type I cytoskeletal 24	1	0.007462271
K1C40_RAT	Keratin, type I cytoskeletal 40	1	0.005774631
K22E_RAT	Keratin, type II cytoskeletal 2 epidermal	1	0.005756503
LDHB_RAT	L-lactate dehydrogenase B chain	1	0.009730358
LDHC_RAT	L-lactate dehydrogenase C chain	1	0.005216281
LPPRC_RAT	Leucine-rich PPR motif-containing protein, mitochondrial	1	0.001244309
MED23_RAT	Mediator of RNA polymerase II transcription subunit 23	1	0.003671483
MP2K5_RAT	Dual specificity mitogen-activated protein kinase kinase 5	1	0.00486131
MYL4_RAT	Myosin light chain 4	1	0.004393665
PDIA1_RAT	Protein disulfide-isomerase	1	0.008339707
PDIA3_RAT	Protein disulfide-isomerase A3	1	0.007027424
PROF1_RAT	Profilin-1	1	0.004634507
PYGB_RAT	Glycogen phosphorylase, brain form (Fragment)	1	0.007993329
PYGL_RAT	Glycogen phosphorylase, liver form	1	0.008605459
RB11A_RAT	Ras-related protein Rab-11A	1	0.00919368
RNZ2_RAT	Zinc phosphodiesterase ELAC protein 2	1	0.009931905
SO1A3_RAT	Solute carrier organic anion transporter family member 1A3	1	0.007670778
SODC_RAT	Superoxide dismutase [Cu-Zn]	1	0.009215279
SPA3K_RAT	Serine protease inhibitor A3K	1	0.009611257
SPAT1_RAT	Spermatogenesis-associated protein 1	1	0.009310239

Protein accession	Protein name	Fold change	p-value
TBB2A_RAT	Tubulin beta-2A chain	1	0.004136669
TBB2C_RAT	Tubulin beta-2C chain	1	0.005183265
TRY1_RAT	Anionic trypsin-1	1	0.004753359
S10A6_RAT	Protein S100-A6	1	0.004064956
ACTA_RAT	Actin, aortic smooth muscle	0.96013289	0.004551115
ENOB_RAT	Beta-enolase	0.96	0.001922148
K2C4_RAT	Keratin, type II cytoskeletal 4	0.924242424	0.000260926
CERU_RAT	Ceruloplasmin	0.923076923	0.000903168
ACTH_RAT	Actin, gamma-enteric smooth muscle	0.842403628	0.003961641
MYH7_RAT	Myosin-7	0.84057971	0.001529886
ENOG_RAT	Gamma-enolase	0.822222222	0.002063611
FIBG_RAT	Fibrinogen gamma chain	0.774193548	0.009256218
ACTC_RAT	Actin, alpha cardiac muscle 1	0.753913043	0.005059552
KNT2_RAT	T-kininogen 2	0.740740741	0.00558715
SERPH_RAT	Serpin H1	0.733333333	0.009921355
S10A9_RAT	Protein S100-A9	0.732984293	0.008013658
ACTS_RAT	Actin, alpha skeletal muscle	0.731355932	0.005625818
K1C19_RAT	Keratin, type I cytoskeletal 19	0.712871287	0.003665853
H2A1_RAT	Histone H2A type 1	0.700507614	0.004178636
H2B1_RAT	Histone H2B type 1	0.679970436	0.006975321
ANXA2_RAT	Annexin A2	0.666666667	0.008850629
ENOA_RAT	Alpha-enolase	0.652173913	0.00322862
ACTG_RAT	Actin, cytoplasmic 2	0.640077821	0.007277743
PIP_RAT	Prolactin-inducible protein homolog	0.636363636	0.0045018
VTDB_RAT	Vitamin D-binding protein	0.631578947	0.004821198
MYL6_RAT	Myosin light polypeptide 6	0.625	0.007716912
RLA1_RAT	60S acidic ribosomal protein P1	0.606060606	0.00961844
K2C1_RAT	Keratin, type II cytoskeletal 1	0.605128205	0.009290293
ITPR3_RAT	Inositol 1,4,5-trisphosphate receptor type 3	0.6	0.000415923
VIME_RAT	Vimentin	0.582677165	0.00602915
CO1A2_RAT	Collagen alpha-2(I) chain	0.580645161	0.002029249
SEBP2_RAT	Selenocysteine insertion sequence-binding protein 2	0.571428571	0.009289995
TPM2_RAT	Tropomyosin beta chain	0.524675325	0.007963654
FRIL1_RAT	Ferritin light chain 1	0.517985612	0.007054747
UBIQ_RAT	Ubiquitin	0.502538071	0.004627577
FIBA_RAT	Fibrinogen alpha chain	0.5	0.006487696
ANXA5_RAT	Annexin A5	0.473684211	0.006577084
FIBB_RAT	Fibrinogen beta chain	0.461538462	0.007610509
HSPB1_RAT	Heat shock protein beta-1	0.454545455	0.007795382
LYSC1_RAT	Lysozyme C-1	0.441481481	0.009485108
ANXA1_RAT	Annexin A1	0.433333333	0.009442622
H2A1C_RAT	Histone H2A type 1-C	0.428838951	0.005777053
G3P_RAT	Glyceraldehyde-3-phosphate dehydrogenase	0.413461538	0.002136295
MYL1_RAT	Myosin light chain 1/3, skeletal muscle isoform	0.39031339	0.009270148
PPIA_RAT	Peptidyl-prolyl cis-trans isomerase A	0.37976438	0.00688846
S10A8_RAT	Protein S100-A8	0.377210884	0.008376005

Protein accession	Protein name	Fold change	p-value
CAH3_RAT	Carbonic anhydrase 3	0.373263889	0.006855583
K2C5_RAT	Keratin, type II cytoskeletal 5	0.362903226	0.001683018
PGAM1_RAT	Phosphoglycerate mutase 1	0.358208955	0.003226668
K1C14_RAT	Keratin, type I cytoskeletal 14	0.357438017	0.003092003
TTHY_RAT	Transthyretin	0.346456693	0.008494551
MYH8_RAT	Myosin-8 (Fragment)	0.345588235	0.009839024
MYH3_RAT	Myosin-3	0.339869281	0.008191661
ALDOA_RAT	Fructose-bisphosphate aldolase A	0.334217507	0.002615368
MYH9_RAT	Myosin-9	0.333333333	2.19223E-05
BRE1B_RAT	E3 ubiquitin-protein ligase BRE1B	0.333333333	0.008763412
K2C75_RAT	Keratin, type II cytoskeletal 75	0.312056738	0.004073721
AT2A1_RAT	Sarcoplasmic/endoplasmic reticulum calcium ATPase 1	0.288461538	0.005508664
KCRM_RAT	Creatine kinase M-type	0.287899861	0.008515449
SCNBA_RAT	Sodium channel protein type 11 subunit alpha	0.285714286	0.008735628
PGAM2_RAT	Phosphoglycerate mutase 2	0.283464567	0.001615161
CO1A1_RAT	Collagen alpha-1(I) chain	0.275362319	0.007271724
MLRS_RAT	Myosin regulatory light chain 2, skeletal muscle isoform	0.226641998	0.007674158
ATPB_RAT	ATP synthase subunit beta, mitochondrial	0.198324022	0.001724103
TNNT3_RAT	Troponin T, fast skeletal muscle	0.1875	6.60958E-05
MAGI3_RAT	Membrane-associated guanylate kinase, WW and PDZ domain-containing protein 3	0.181818182	0.005561035
MYH4_RAT	Myosin-4	0.177721088	0.00895155
KPYR_RAT	Pyruvate kinase isozymes R/L	0.172413793	0.008900754
TEP1_RAT	Telomerase protein component 1	0.166666667	0.002576875
APOE_RAT	Apolipoprotein E	0.138297872	0.002788608
UBR4_RAT	E3 ubiquitin-protein ligase UBR4	0.111111111	0.001928186
FRAP_RAT	Serine/threonine-protein kinase mTOR	0.044444444	0.002014783
MYH6_RAT	Myosin-6	0.04265873	0.006292763
