# Supplementary material for: Sericin coated thin polymeric films reduce keratinocyte proliferation via the mTOR pathway and epidermal inflammation through IL17 signaling in psoriasis rat model
Source: Sci Rep. 2023 Jul 26;13:12133. doi: 10.1038/s41598-023-39218-y (PMC10372088; doi:10.1038/s41598-023-39218-y)
Supplement: Supplementary file 4 — Supplementary Table S4. [file 41598_2023_39218_MOESM4_ESM.pdf]

Table S4. The 157 significant alteration proteins from 1,269 co-observational proteins divided by down-regulation.

Protein accession	Protein name	Fold change	p-value
MYH6_RAT	Myosin-6	23.44186047	0.006292763
FRAP_RAT	Serine/threonine-protein kinase mTOR	22.5	0.002014783
UBR4_RAT	E3 ubiquitin-protein ligase UBR4	9	0.001928186
APOE_RAT	Apolipoprotein E	7.230769231	0.002788608
TEP1_RAT	Telomerase protein component 1	6	0.002576875
KPYR_RAT	Pyruvate kinase isozymes R/L	5.8	0.008900754
MYH4_RAT	Myosin-4	5.626794258	0.00895155
MAGI3_RAT	Membrane-associated guanylate kinase, WW and PDZ domain-containing protein 3	5.5	0.005561035
TNNT3_RAT	Troponin T, fast skeletal muscle	5.333333333	6.60958E-05
ATPB_RAT	ATP synthase subunit beta, mitochondrial	5.042253521	0.001724103
MLRS_RAT	Myosin regulatory light chain 2, skeletal muscle isoform	4.412244898	0.007674158
CO1A1_RAT	Collagen alpha-1(I) chain	3.631578947	0.007271724
PGAM2_RAT	Phosphoglycerate mutase 2	3.527777778	0.001615161
SCNBA_RAT	Sodium channel protein type 11 subunit alpha	3.5	0.008735628
KCRM_RAT	Creatine kinase M-type	3.473429952	0.008515449
AT2A1_RAT	Sarcoplasmic/endoplasmic reticulum calcium ATPase 1	3.466666667	0.005508664
K2C75_RAT	Keratin, type II cytoskeletal 75	3.204545455	0.004073721
BRE1B_RAT	E3 ubiquitin-protein ligase BRE1B	3	0.008763412
MYH9_RAT	Myosin-9	3	2.19223E-05
ALDOA_RAT	Fructose-bisphosphate aldolase A	2.992063492	0.002615368
MYH3_RAT	Myosin-3	2.942307692	0.008191661
MYH8_RAT	Myosin-8 (Fragment)	2.893617021	0.009839024
TTHY_RAT	Transthyretin	2.886363636	0.008494551
K1C14_RAT	Keratin, type I cytoskeletal 14	2.797687861	0.003092003
PGAM1_RAT	Phosphoglycerate mutase 1	2.791666667	0.003226668
K2C5_RAT	Keratin, type II cytoskeletal 5	2.755555556	0.001683018
CAH3_RAT	Carbonic anhydrase 3	2.679069767	0.006855583
S10A8_RAT	Protein S100-A8	2.65103697	0.008376005
PPIA_RAT	Peptidyl-prolyl cis-trans isomerase A	2.633211679	0.00688846
MYL1_RAT	Myosin light chain 1/3, skeletal muscle isoform	2.562043796	0.009270148
G3P_RAT	Glyceraldehyde-3-phosphate dehydrogenase	2.418604651	0.002136295
H2A1C_RAT	Histone H2A type 1-C	2.331877729	0.005777053
ANXA1_RAT	Annexin A1	2.307692308	0.009442622
LYSC1_RAT	Lysozyme C-1	2.265100671	0.009485108
HSPB1_RAT	Heat shock protein beta-1	2.2	0.007795382
FIBB_RAT	Fibrinogen beta chain	2.166666667	0.007610509
ANXA5_RAT	Annexin A5	2.111111111	0.006577084
FIBA_RAT	Fibrinogen alpha chain	2	0.006487696
UBIQ_RAT	Ubiquitin	1.98989899	0.004627577
FRIL1_RAT	Ferritin light chain 1	1.930555556	0.007054747
TPM2_RAT	Tropomyosin beta chain	1.905940594	0.007963654

Protein accession	Protein name	Fold change	p-value
SEBP2_RAT	Selenocysteine insertion sequence-binding protein 2	1.75	0.009289995
CO1A2_RAT	Collagen alpha-2(I) chain	1.722222222	0.002029249
VIME_RAT	Vimentin	1.716216216	0.00602915
ITPR3_RAT	Inositol 1,4,5-trisphosphate receptor type 3	1.666666667	0.000415923
K2C1_RAT	Keratin, type II cytoskeletal 1	1.652542373	0.009290293
RLA1_RAT	60S acidic ribosomal protein P1	1.65	0.00961844
MYL6_RAT	Myosin light polypeptide 6	1.6	0.007716912
VTDB_RAT	Vitamin D-binding protein	1.583333333	0.004821198
PIP_RAT	Prolactin-inducible protein homolog	1.571428571	0.0045018
ACTG_RAT	Actin, cytoplasmic 2	1.56231003	0.007277743
ENOA_RAT	Alpha-enolase	1.533333333	0.00322862
ANXA2_RAT	Annexin A2	1.5	0.008850629
H2B1_RAT	Histone H2B type 1	1.470652174	0.006975321
H2A1_RAT	Histone H2A type 1	1.427536232	0.004178636
K1C19_RAT	Keratin, type I cytoskeletal 19	1.402777778	0.003665853
ACTS_RAT	Actin, alpha skeletal muscle	1.367323291	0.005625818
S10A9_RAT	Protein S100-A9	1.364285714	0.008013658
SERPH_RAT	Serpin H1	1.363636364	0.009921355
KNT2_RAT	T-kininogen 2	1.35	0.00558715
ACTC_RAT	Actin, alpha cardiac muscle 1	1.326412918	0.005059552
FIBG_RAT	Fibrinogen gamma chain	1.291666667	0.009256218
ENOG_RAT	Gamma-enolase	1.216216216	0.002063611
MYH7_RAT	Myosin-7	1.189655172	0.001529886
ACTH_RAT	Actin, gamma-enteric smooth muscle	1.187079408	0.003961641
CERU_RAT	Ceruloplasmin	1.083333333	0.000903168
K2C4_RAT	Keratin, type II cytoskeletal 4	1.081967213	0.000260926
ENOB_RAT	Beta-enolase	1.041666667	0.001922148
ACTA_RAT	Actin, aortic smooth muscle	1.041522491	0.004551115
S10A6_RAT	Protein S100-A6	1	0.004064956
ATP5H_RAT	ATP synthase subunit d, mitochondrial	1	0.004753359
BCAR1_RAT	Breast cancer anti-estrogen resistance protein 1	1	0.005183265
CASQ1_RAT	Calsequestrin-1 (Fragment)	1	0.004136669
CBR1_RAT	Carbonyl reductase [NADPH] 1	1	0.009310239
CRP_RAT	C-reactive protein	1	0.009611257
CYTA_RAT	Cystatin-A	1	0.009215279
DESM_RAT	Desmin	1	0.007670778
EF2_RAT	Elongation factor 2	1	0.009931905
FABP5_RAT	Fatty acid-binding protein, epidermal	1	0.00919368
FABPH_RAT	Fatty acid-binding protein, heart	1	0.008605459
FGFR4_RAT	Fibroblast growth factor receptor 4	1	0.007993329
FINC_RAT	Fibronectin	1	0.004634507
FUBP1_RAT	Far upstream element-binding protein 1	1	0.007027424
GNAS1_RAT	Guanine nucleotide-binding protein G(s) subunit alpha isoforms XLas	1	0.008339707
ITAD_RAT	Integrin alpha-D	1	0.004393665
K1C12_RAT	Keratin, type I cytoskeletal 12	1	0.00486131
K1C18_RAT	Keratin, type I cytoskeletal 18	1	0.003671483

Protein accession	Protein name	Fold change	p-value
K1C24_RAT	Keratin, type I cytoskeletal 24	1	0.001244309
K1C40_RAT	Keratin, type I cytoskeletal 40	1	0.005216281
K22E_RAT	Keratin, type II cytoskeletal 2 epidermal	1	0.009730358
LDHB_RAT	L-lactate dehydrogenase B chain	1	0.005756503
LDHC_RAT	L-lactate dehydrogenase C chain	1	0.005774631
LPPRC_RAT	Leucine-rich PPR motif-containing protein, mitochondrial	1	0.007462271
MED23_RAT	Mediator of RNA polymerase II transcription subunit 23	1	0.00573286
MP2K5_RAT	Dual specificity mitogen-activated protein kinase kinase 5	1	0.005109198
MYL4_RAT	Myosin light chain 4	1	0.005078197
PDIA1_RAT	Protein disulfide-isomerase	1	0.008964533
PDIA3_RAT	Protein disulfide-isomerase A3	1	0.002904278
PROF1_RAT	Profilin-1	1	0.006957035
PYGB_RAT	Glycogen phosphorylase, brain form (Fragment)	1	0.005152272
PYGL_RAT	Glycogen phosphorylase, liver form	1	0.007165056
RB11A_RAT	Ras-related protein Rab-11A	1	0.004910671
RNZ2_RAT	Zinc phosphodiesterase ELAC protein 2	1	0.002156517
SO1A3_RAT	Solute carrier organic anion transporter family member 1A3	1	0.006837389
SODC_RAT	Superoxide dismutase [Cu-Zn]	1	0.009967723
SPA3K_RAT	Serine protease inhibitor A3K	1	0.004566037
SPAT1_RAT	Spermatogenesis-associated protein 1	1	0.008450625
TBB2A_RAT	Tubulin beta-2A chain	1	0.008427163
TBB2C_RAT	Tubulin beta-2C chain	1	0.007270457
TRY1_RAT	Anionic trypsin-1	1	0.003436997
APOH_RAT	Beta-2-glycoprotein 1	1	0.005000915
DDX52_RAT	Probable ATP-dependent RNA helicase DDX52	1	0.002106268
ECHD3_RAT	Enoyl-CoA hydratase domain-containing protein 3, mitochondrial	1	0.004992832
H12_RAT	Histone H1.2	0.981481481	0.004130336
HBA_RAT	Hemoglobin subunit alpha-1/2	0.974477958	0.004581799
TPM3_RAT	Tropomyosin alpha-3 chain	0.972972973	0.005000702
TPIS_RAT	Triosephosphate isomerase	0.937915743	0.003675832
KNT1_RAT	T-kininogen 1	0.931034483	0.004169749
K2C1B_RAT	Keratin, type II cytoskeletal 1b	0.9	0.005567882
DYN3_RAT	Dynamin-3	0.888888889	0.007446465
K1C15_RAT	Keratin, type I cytoskeletal 15	0.879518072	0.001121454
LDHA_RAT	L-lactate dehydrogenase A chain	0.854368932	0.009429266
ALBU_RAT	Serum albumin	0.850571257	0.003086273
KPYM_RAT	Pyruvate kinase isozymes M1/M2	0.76	7.53773E-05
FETUA_RAT	Alpha-2-HS-glycoprotein	0.75	0.003775264
K1C13_RAT	Keratin, type I cytoskeletal 13	0.75	0.002906459
K1C42_RAT	Keratin, type I cytoskeletal 42	0.736842105	0.003379619
K2C73_RAT	Keratin, type II cytoskeletal 73	0.727272727	0.007756567

Protein accession	Protein name	Fold change	p-value
H1T_RAT	Histone H1t	0.714285714	0.003371031
H4_RAT	Histone H4	0.704845815	0.002877408
TPM1_RAT	Tropomyosin alpha-1 chain	0.671428571	0.002799989
PPARG_RAT	Peroxisome proliferator-activated receptor gamma	0.666666667	0.002052374
H31_RAT	Histone H3.1	0.636363636	0.003223036
K2C6A_RAT	Keratin, type II cytoskeletal 6A	0.636217949	1.18396E-06
CO9_RAT	Complement component C9	0.625	0.009328321
MCPT1_RAT	Mast cell protease 1	0.609022556	0.003558385
HSP7C_RAT	Heat shock cognate 71 kDa protein	0.6	0.006381825
APOA1_RAT	Apolipoprotein A-I	0.593023256	0.003154238
TPM4_RAT	Tropomyosin alpha-4 chain	0.580645161	0.0025809
K1C10_RAT	Keratin, type I cytoskeletal 10	0.576923077	0.003607441
HSP72_RAT	Heat shock-related 70 kDa protein 2	0.5	0.009858252
HBB1_RAT	Hemoglobin subunit beta-1	0.47752809	0.002681579
HEMO_RAT	Hemopexin	0.469387755	0.001931977
A1M_RAT	Alpha-1-macroglobulin	0.45	0.001155084
PYGM_RAT	Glycogen phosphorylase, muscle form	0.428571429	0.008760692
TRFE_RAT	Serotransferrin	0.380136986	0.000918597
HBB2_RAT	Hemoglobin subunit beta-2	0.375576037	0.006562916
PGK1_RAT	Phosphoglycerate kinase 1	0.358974359	0.002952805
SPA3N_RAT	Serine protease inhibitor A3N	0.344262295	0.000870061
LUM_RAT	Lumican	0.343220339	0.001327189
MDHC_RAT	Malate dehydrogenase, cytoplasmic	0.341772152	0.000731377
A1AT_RAT	Alpha-1-antiproteinase	0.319148936	0.006772844
A1BG_RAT	Alpha-1B-glycoprotein	0.302325581	0.001640713
LMNA_RAT	Lamin-A	0.285714286	0.008154672
CO3_RAT	Complement C3	0.243243243	0.000275365
PGS2_RAT	Decorin	0.230769231	0.000341035
H2A4_RAT	Histone H2A type 4	0.1	0.004738036
